# Synthesis of 1,2,3,4‐Tetrahydroisoquinoline‐3‐Carboxylic Acid‐Embedded Decapeptides: In Silico Studies and In Vitro Evaluation Against Breast Cancer

**DOI:** 10.1002/open.202500282

**Published:** 2025-12-17

**Authors:** Sunil Tivari, Naurin Lalani, Pritam Bagwe, Prashant Chandole, Ramesh Pawar, Enrique Delgado‐Alvarado, Abdallah Othman Ghnaim, Yashwantsinh Jadeja

**Affiliations:** ^1^ Department of Chemistry Marwadi University Rajkot India; ^2^ Department of Pharmaceutical Sciences and Technology Institute of Chemical Technology Mumbai India; ^3^ Micro and Nanotechnology Research Center Universidad Veracruzana Boca del Río Mexico; ^4^ Department of Chemistry Faculty of Science Al‐Balqa′ Applied University Salt Jordan

**Keywords:** amphipathic peptides, antiproliferation, breast cancer, peptides, tetrahydroisoquinoline

## Abstract

Cancer is a leading cause of mortality worldwide, which has led to further research in developing more effective therapeutics. We synthesized amphipathic cationic peptides incorporating 1,2,3,4‐tetrahydroisoquinoline‐3‐carboxylic acid (Tic acid) into their backbones to explore their potential anticancer properties. The MCF‐7 cell line was selected to evaluate cell viability, inhibition percentage, and cytotoxic effects of the synthesized decapeptides. The results suggested DEC‐1 (IC_50_ = 3.38 µM) to be the most potent candidate, showcasing antiproliferative activity similar to the standard drug tamoxifen (IC_50_ = 2.68 µM). Structural characterization of the peptides confirmed the successful incorporation of Tic into the peptide backbones. The peptides were docked with xanthine oxidase (PDB ID: 2CKJ) and Nrf2 (Nuclear factor erythroid 2‐related factor 2) inhibitor protein Keap‐1 (PDB ID: 7Q6S), revealing strong binding interactions, particularly for DEC‐1, having a binding score of nearly −8.864 kcal/mol—which is stronger than its reference ligand with Keap‐1 protein—suggesting possible inhibitory roles in cancer cell proliferation and oxidative stress regulation. These promising findings indicate that the potent molecule DEC‐1 can be taken for further studies and might lead to a potential therapeutic agent for cancer treatment.

## Graphical abstract

Synthesized decapeptides were designed and validated by in silico studies. They have significantly exhibited anticancer potential against breast cancer and MCF‐7 cell line. The above illustration specifies the synthetic pathway, biological screening, and the mode of action of the peptide in blocking the pathways responsible for the growth of cancer cells.

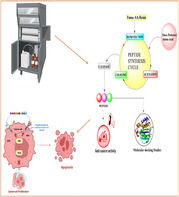

## 1 Introduction

In the 21st century, cancer has emerged as a prominent cause of mortality across the globe [1, 2]. Cancer treatments either include radiation therapy or chemotherapy, which have significant adverse effects on the health of the patient and the cells can become resistant to cancer medications [3, 4, 5]. To overcome the current situation, there is the utmost need to develop novel anticancer therapeutics having lesser toxicity, high plasma stability, and an amphipathic nature so that they can easily cross the cell membrane and can hold back the disease to its maximum potential.

**FIGURE 1:**
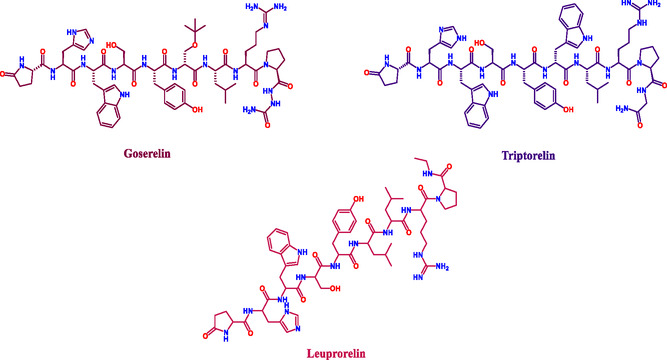
Commercially available peptide‐based drugs for the treatment of breast cancer.

Peptide‐based anticancer drugs selectively bind to receptors such as epidermal growth factor receptor (EGFR), human EGFR2 (HER2), or integrins, interfering with key signaling pathways [6, 7, 8]. They are often overexpressed in breast cancer and lead to activation of downstream signaling pathways like PI3K/Akt and MAPK, promoting cell survival and proliferation [9, 10, 11, 12]. This targeted action triggers apoptosis, inhibits angiogenesis, and minimizes toxicity, ensuring effective cancer cell elimination while sparing healthy tissues [7, 13, 14]. Commercially available drugs for the treatment of cancer, such as goserelin, triptorelin, and leuprorelin, are peptide‐based therapeutics for treating breast cancer (Figure 1).

**FIGURE 2:**
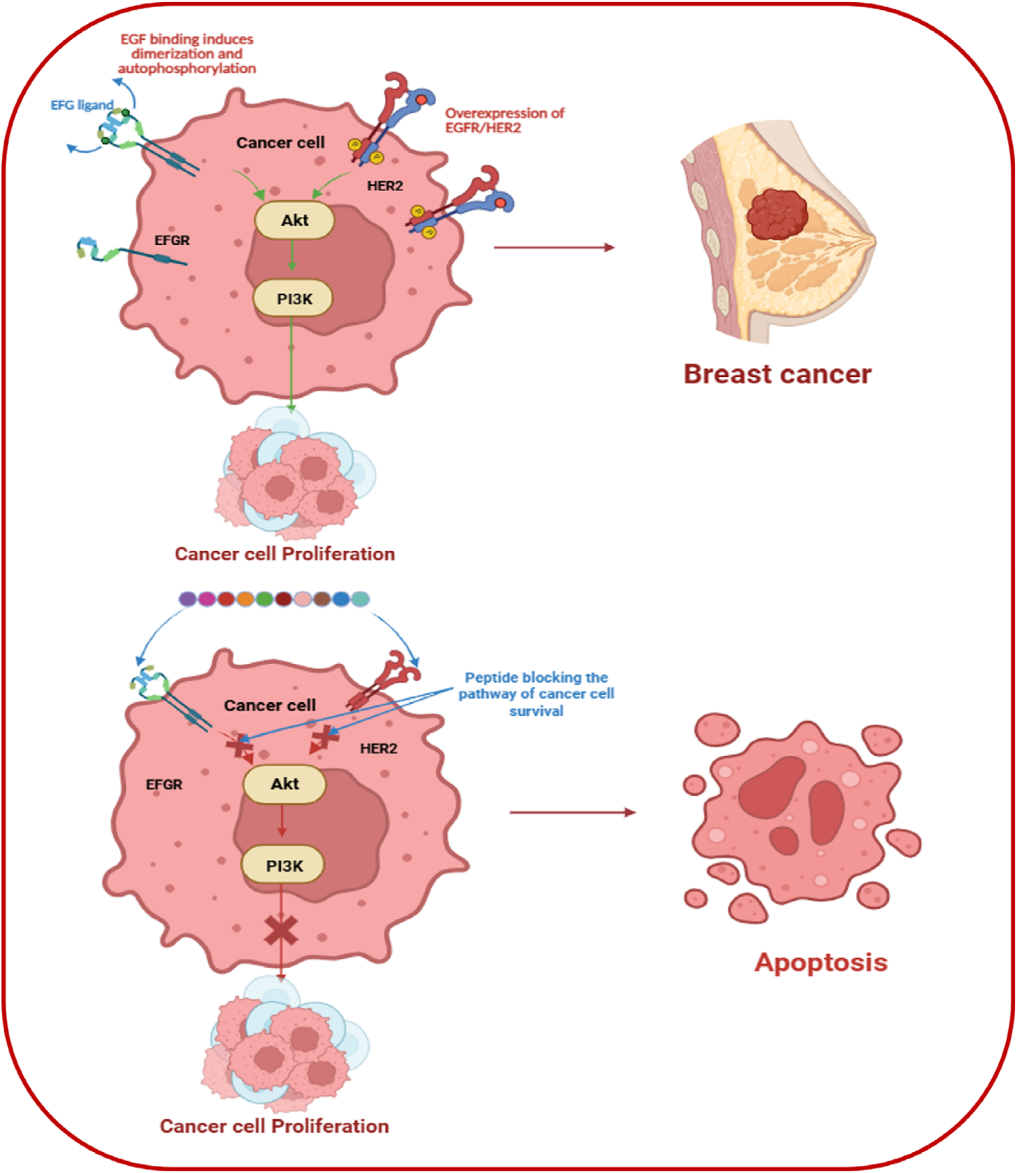
Mode of action of peptide therapeutics in breast cancer. Anticancer peptide is shown for the blocking the pathways which inhibits Akt signaling and leads to disruption mechanism of cancer cells. (Created by Biorender.com).

Amphipathic cationic peptides have proven to be a potential group of bioactive molecules exhibiting potent anticancer properties [15, 16] and have attracted considerable attention due to their characteristic combination of hydrophilic and hydrophobic regions, allowing them to disrupt cell membranes effectively [17, 18, 19, 20, 21]. Modern research has unveiled the selective anticancer properties of amphipathic cationic peptides, which selectively target cancer cells, safeguarding healthy cells [22, 23, 24] and triggering apoptotic pathways or disrupting membrane integrity (Figure 2) [10, 25]. This dual functionality makes them favorable candidates for cancer intervention with reduced side effects, addressing the growing need for more targeted and less toxic cancer treatments [10, 26, 27].

The rationale behind our work was designing peptide chains having unnatural amino acids and heterocyclic side chains which have biological potency, and Tic acid is hydrophobic, rigid, and aromatic in nature and mimics the amino acids like phenylalanine and proline, which introduces conformational constraint and increases the stability. Tic acid exhibits amphipathic nature, which can directly induce cell disruption and pore formation [28, 29, 30]. The estrogen receptor‐positive (ER+) MCF‐7 cell line was selected for in vitro studies of the decapeptides. The selection of the MCF‐7 cell line was based on its well‐documented response to chemotherapeutic agents and its relevance in evaluating potential anticancer peptides targeting ER+, making it representative of hormone‐responsive breast cancer, which is the main focus of the study [31, 32]. The designed series exhibited selective cytotoxic effects on the MCF‐7 breast cancer cells [33, 34, 35]. For in silico studies, xanthine oxidase (XO) (PDB ID:2CKJ) and Keap‐1 (PDB ID:7Q6S) were taken. The results show that peptides may reduce reactive oxygen species (ROSs) by targeting and inhibiting the active site of XO. The peptide molecule may disrupt keap‐1‐Nrf2 interaction, which helps in stabilizing Nrf2, promoting antioxidant activity and limiting breast cancer progression.

The protein structures for Keap‐1 and XO were selected for the in silico docking studies based on their established roles in oxidative stress and breast cancer pathophysiology. Keap‐1 is a key regulator of the Nrf2 antioxidant pathway, while XO contributes to ROS generation and tumor progression. Both crystal structures contain cocrystallized ligands in the active site, ensuring accurate definition of the binding pocket during grid generation. These structures also exhibit high sequence coverage and minimal missing residues, closely matching the natural protein sequences, which improves docking reliability. The selection criteria thus combined biological relevance, structural resolution, and ligand‐defined binding site information to ensure robust molecular docking analysis.

This article delves into the synthesis, characterization, and activity of these peptides, aiming to contribute to the development of next‐generation therapeutics with the potential to revolutionize medicine and impact global health outcomes. Continued research and advancements in this field have the potential to reshape the landscape of modern medicine and significantly impact global health outcomes.

## 2 Results and Discussion

### 2.1 Chemistry

Developing the amphipathic peptides and conjugating with heterocyclic molecule can enhance its biological potency by improving the solubility in aqueous medium and can improve the stability of the compound [36, 37]. Selectively incorporating conformationally constrained amino acid units into a peptide chain is a useful strategy to produce metabolically stable peptides with improved receptor selectivity [38, 39]. The identification and selection of amino acids is based on the characteristics of specific functional groups and side chains of amino acids as they can modulate key cellular processes.

To effectively target breast cancer cells, the peptide was designed using specific amino acids that offer both functional and structural advantages. Histidine (His) was selected for its role in coordinating metal ions and participating in intracellular enzyme functions [40]. Glycine (Gly) was added to maintain backbone flexibility, allowing the peptide to adjust to the tumor microenvironment [41]. The inclusion of arginine (Arg) and lysine (Lys), both carrying positive charges, enhances interaction with negatively charged membranes of cancer cells, thereby supporting cellular uptake. Glutamic acid (Glu) aids in stabilizing interactions through hydrogen bonding, while serine (Ser), with its hydroxyl group, contributes to molecular recognition [42]. Tryptophan (Trp), being aromatic, supports hydrophobic interactions with membrane components, improving cell selectivity [43]. Cysteine (Cys) offers thiol groups for disulfide bond formation, which increases structural stability [44]. Moreover, Tic acid (1,2,3,4‐tetrahydroisoquinoline‐3‐carboxylic acid) was incorporated to enhance conformational rigidity, improve membrane permeability, and promote receptor‐specific interactions, indirectly modulating estrogen signaling and suppressing hormone‐dependent cell growth [45].

**SCHEME 1:**
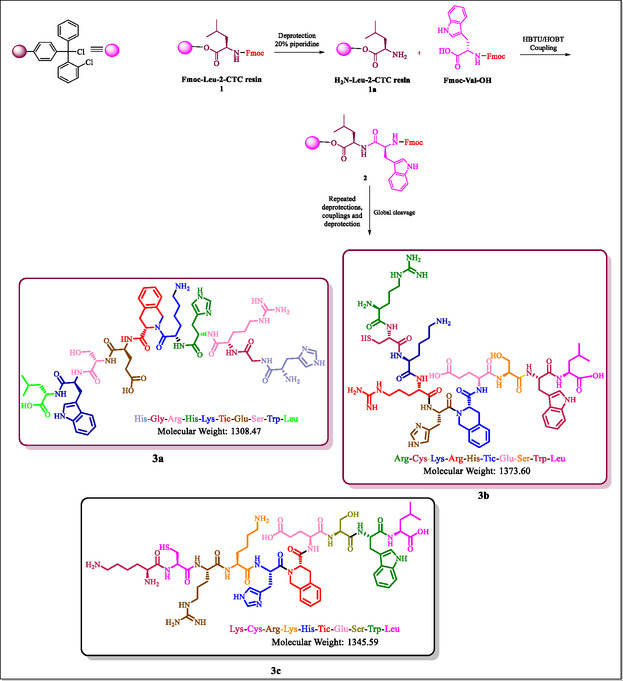
Synthetic route of amphipathic cationic peptides 3a–c.

Decapeptides (Scheme 1) consisting of Tic acid incorporation were designed, and the synthesis of the designed decapeptide was carried out by following solid‐phase peptide synthesis (SPPS) coupling protocol (HBTU/HOBt). The yields synthesized compounds were within the range of 89%–92%. The synthesized peptide derivatives were isolated in the form of solid powder and exhibited exceptional physical stability. The synthesis process is composed of repetitive cycles of coupling followed by deprotection. The completion of the reaction was confirmed by the Kaiser test. The coupling of different amino acids (Fmoc‐R‐OH) was carried out by following the coupling reaction protocol of SPPS using HBTU/HOBt as coupling agents (Scheme 1). Here, “R” refers to the different amino acids. The completion of the reaction is determined by the Kaiser test. The crude peptides obtained were purified using the mixture of ethyl acetate and hexane, and the purity of all the compounds is >93% as per HPLC data. Yields of the desired compounds 3a–c were between 89% and 92%, and their melting point were recorded between 192ºC and 205°C.

### 2.2 Computational Study

To explore the molecular interactions of the molecules for anticancer potential, protein structures relevant to key breast cancer–associated pathways were selected. The estrogen receptor alpha (ERα) ligand‐binding domain (PDB ID: 7Q6S) was chosen as it plays a crucial role in hormone‐dependent cell proliferation, serving as a primary therapeutic target in estrogen‐responsive malignancies. Additionally, XO (PDB ID: 2CKJ) was selected due to its involvement in the generation of ROS, which contribute to oxidative stress–mediated tumor progression. Docking these peptides with both targets enables the evaluation of their potential to modulate estrogen signaling and oxidative pathways associated with cancer growth and survival.

**FIGURE 3:**
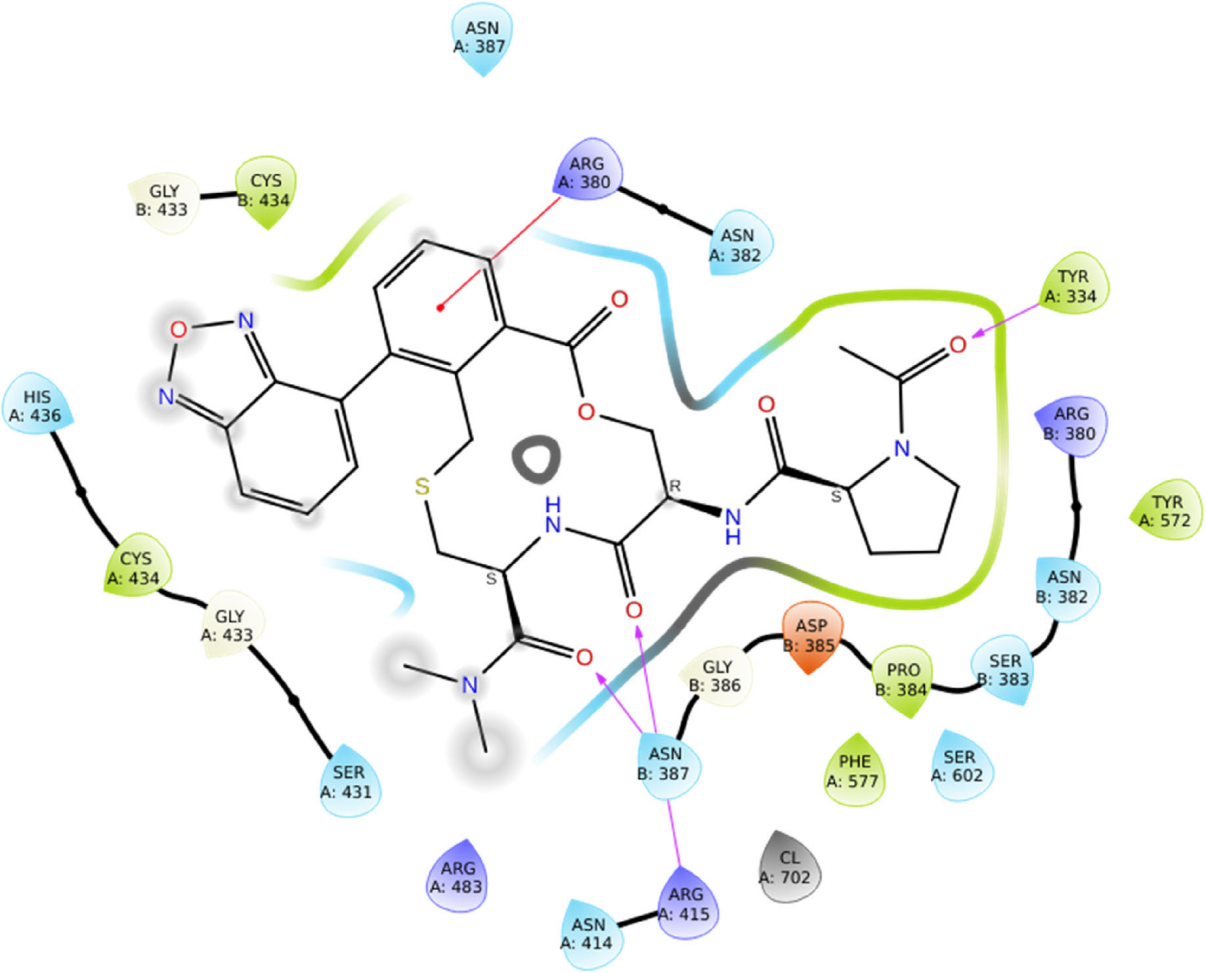
2D interactions of reference molecule (91M) with Keap‐1 (7Q6S).

**FIGURE 4:**
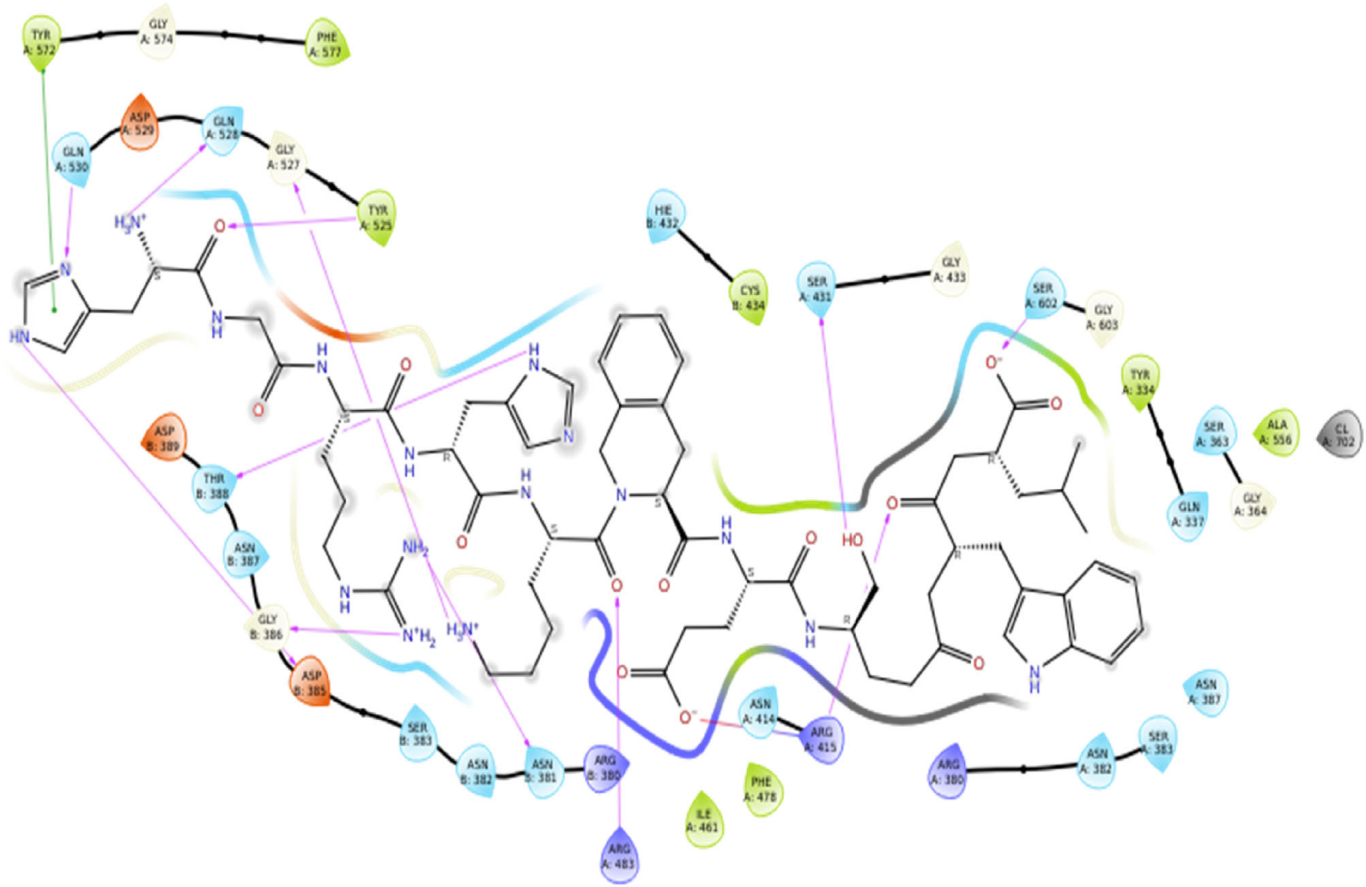
2D interactions of DEC‐1 with Keap‐1 (7Q6S).

With the Keap1 receptor, using the structure (PDB ID: 7Q6S) at a resolution of 2.14 Å, the results indicate that DEC‐1 exhibits superior binding interactions compared to the reference ligand (Figure 3). The results show the active participation of aromatic and polar residues, TYR572 and TYR525, which stabilizes the ligand interactions. GLN530, GLN528, and GLY527 contribute to hydrogen bonding, which stabilizes the ligand orientation. ASN381 and GLY386 shows polar interactions, results in ligand affinity. ARG483, ARG415, and ARG414 being positively charged, form electrostatic and hydrogen bond interactions. SER431 and SER602, along with THR388, being polar residues, which contributes to ligand interactions and hydrogen bonding (Figure 4).

**FIGURE 5:**
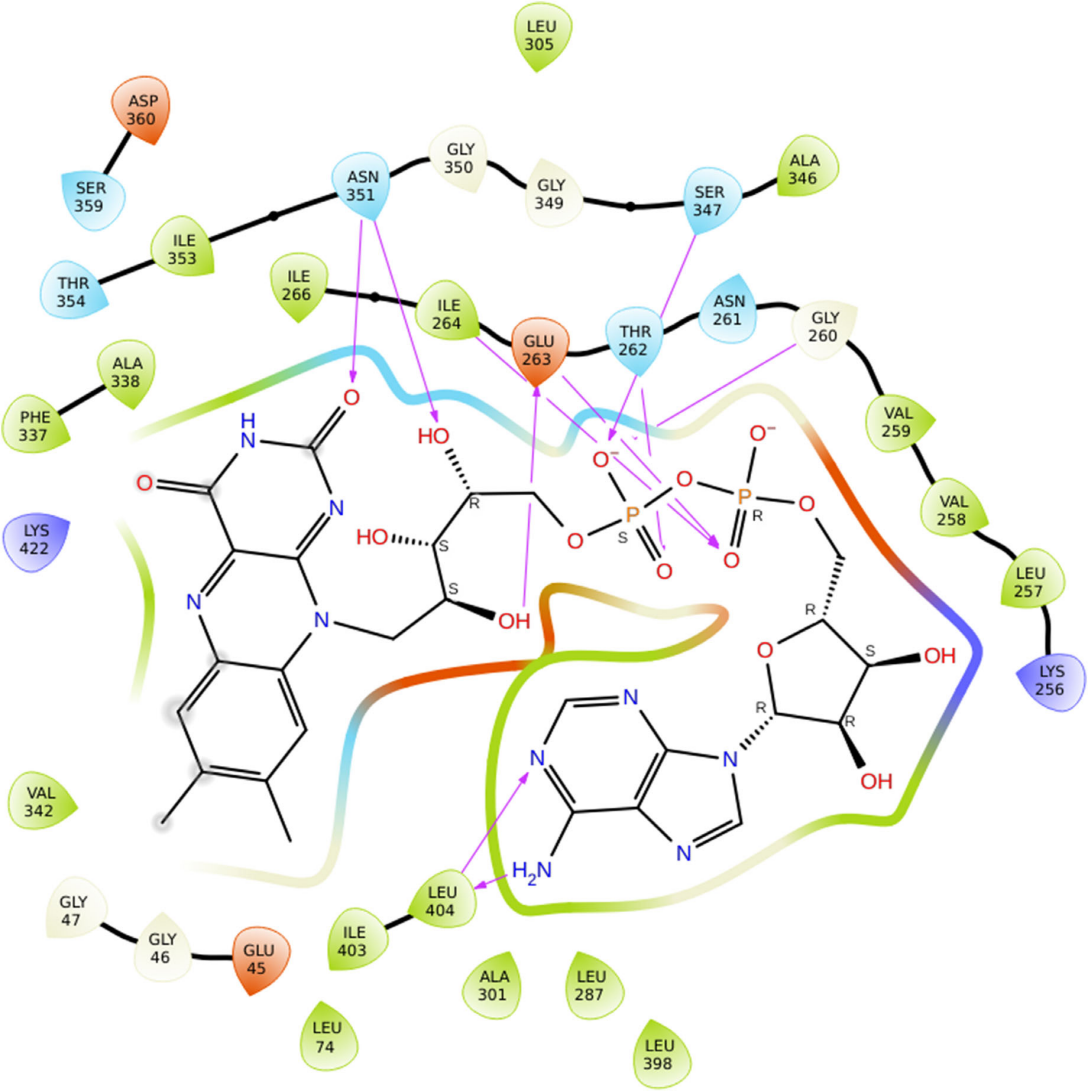
2D interactions of reference molecule (FAD) with XO (2CKJ).

**FIGURE 6:**
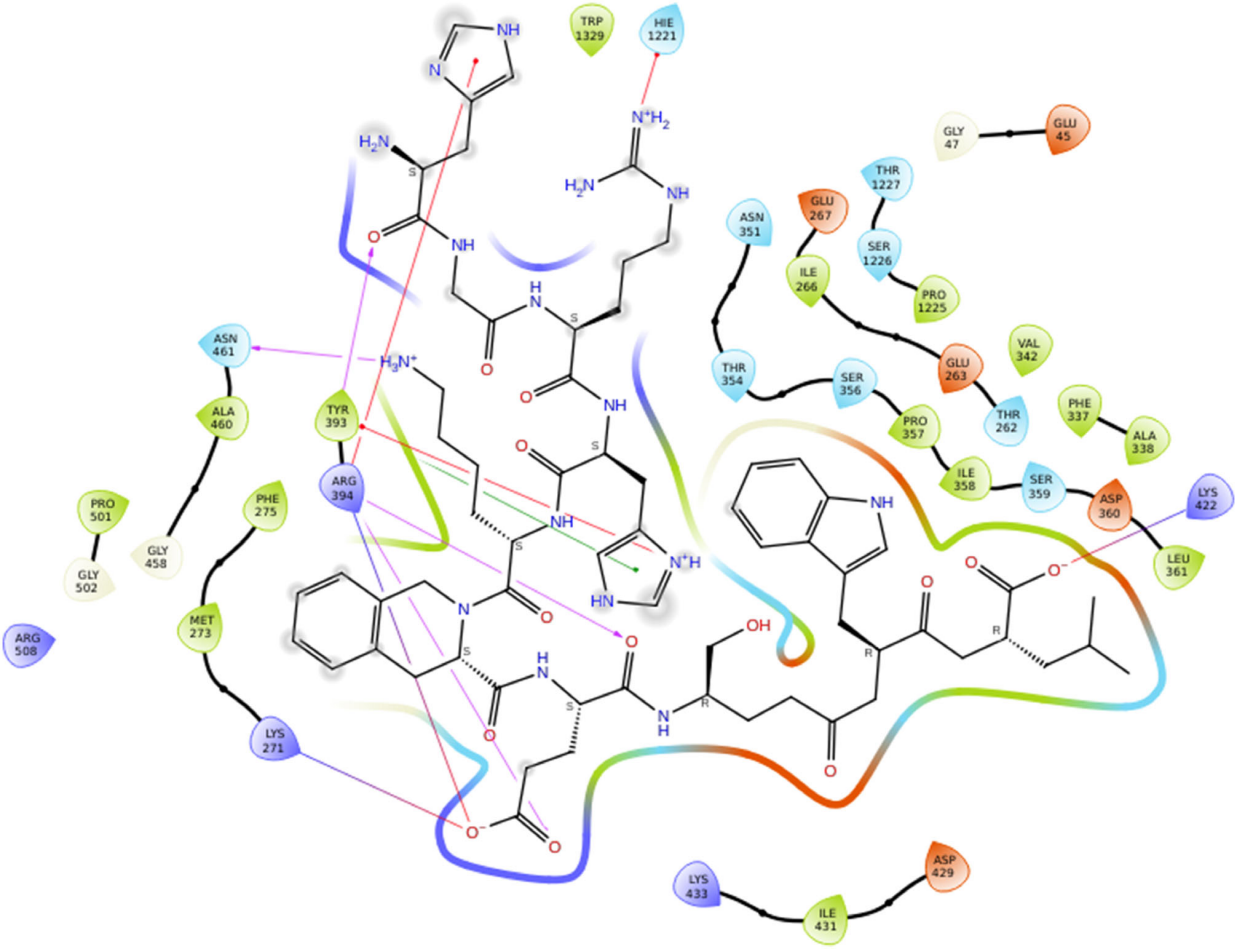
2D interactions of DEC‐1 with XO (2CKJ).

In XO 2CKJ was selected because it had a reference ligand and no mutations reported. Also, the resolution was 3.59 Å. The XP GScore achieved by docking the decapeptides to the XO receptor was comparable to that of the reference ligand (Figure 5), in which DEC‐1 showed superior result. The results suggest that DEC‐1 demonstrated significant hydrogen bonding with residues of XO, such as ARG394, which stabilizes the complex through polar interactions; GLU353, being acidic in nature, helps in ligand anchoring; TYR393 and PHE275 participate in π–π stacking and hydrophobic interactions, which helps in binding affinity and maintaining the pocket shape. MET 421, LEU 346, and LYS 271 provide additional hydrophobic and electrostatic support, reinforcing the binding through van der Waals forces and potential ionic interactions. Since XO is a major source of ROS, its inhibition can reduce oxidative damage and provide protection against ROS‐induced cellular proliferation (Figure 6).

In the docking studies with the regulatory protein Keap‐1 (Table 1), DEC‐1 exhibited a higher docking score (−8.864 kcal/mol) in comparison to the other two peptides. Interestingly, DEC‐1 scored better compared to the reference molecule (91M) (−4.922 Kcal/mol). Also, it is seen that the docking score obtained with the key enzyme XO (Table 2), DEC‐1 (−8.271 Kcal/mol) scored similar binding affinity as reference molecule (FAD) (−10.312 Kcal/mol). It interacts with key residues of the receptor, suggesting that binding to these critical sites could potentially inhibit the enzyme's activity. The docking studies provided insights into the binding interactions and potential efficacy of the ligands against the selected protein target. These findings will guide further optimization and experimental validation of the identified ligands. Further studies will include simulations to validate and refine these findings for enhanced therapeutic insights, which will be considered as a way forward considering the limited scope of this research.

**TABLE 1:** Docking scores of decapeptides Keap‐1 (7Q6S).

Title	Docking score	XP GScore	Glide emodel
Reference ligand (91M)	−4.922	−4.922	−79.42
Decapeptide derivatives			
DEC‐1	−8.864	−10.32	−138.805
DEC‐2	−3.539	−4.833	−98.35
DEC‐3	−5.67	−7.435	−69.524

**TABLE 2:** Docking scores of decapeptides with XO (2CKJ).

Title	Docking score	XP GScore	Glide emodel
Reference ligand (FAD)	−10.312	−10.312	−215.354
Decapeptide derivatives			
DEC‐1	−8.271	−10.385	−143.372
DEC‐2	−2.352	−3.012	−3691.56
DEC‐3	−4.281	−5.294	−126.146

The docking results are given below:

### 2.3 MTT Assay

**FIGURE 7:**
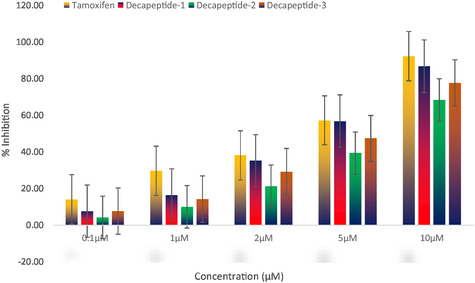
Anticancer activity of decapeptides and the standard drug (tamoxifen) in MCF‐7 cell line determined by MTT assay at different concentrations ranging from 0.1 µM to 10 µM were expressed as mean percentage inhibition (±SD) from three independent experiments (*n* = 3).

The results (Table 3) showed the percentage of inhibition of cell viability (Figure 7) for each compound at different concentrations. At 5 µM, DEC‐1 and tamoxifen exhibited nearly identical % inhibitions, 56.81% and 57.21%, respectively, highlighting DEC‐1's potential as a potent anticancer agent. At the highest concentration of 10 µM, DEC‐1 achieved a % inhibition of 86.73%, close to tamoxifen's 92.21%. DEC‐3 also showed promising results, with 77.56% inhibition at 10 µM, though slightly less potent than DEC‐1.

**TABLE 3:** Cytotoxic activity data (% inhibition and IC_50_ values) of decapeptides with reference to the standard.

Compound	% Inhibition					IC_50_ [µM]
	0.1 µM	1 µM	2 µM	5 µM	10 µM	
Tamoxifen	14.09 ± 5.41	29.62 ± 10.42	38.16 ± 2.76	57.21 ± 5.99	92.21 ± 0.73	2.68
DEC‐1	7.57 ± 1.29	16.37 ± 2.13	35.18 ± 1.36	56.81 ± 2.20	86.74 ± 1.58	3.38
DEC‐2	4.24 ± 0.40	9.98 ± 0.88	21.20 ± 1.73	39.38 ± 1.20	68.28 ± 1.97	6.10
DEC‐3	7.68 ± 0.69	14.23 ± 0.93	29.17 ± 0.59	47.33 ± 2.13	77.56 ± 1.60	4.49

**FIGURE 8:**
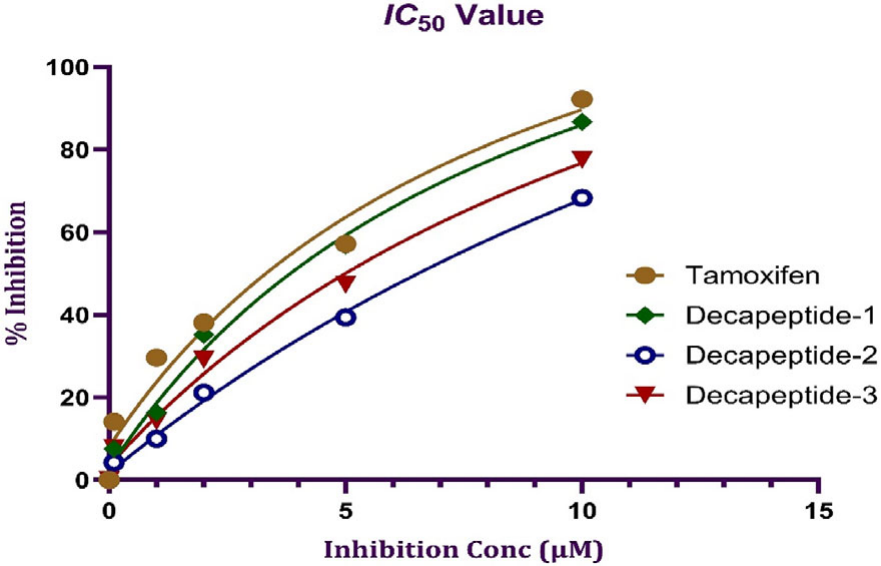
Graphical representation of IC_50_ values at different concentrations ranging from 0.1 to 10 µM.

The standard drug tamoxifen exhibited an IC_50_ value of 2.68 µM. Among the synthesized decapeptides screened for cytotoxicity using the MTT assay, DEC‐1 and DEC‐3 demonstrated significant cytotoxic effects, with IC_50_ values of 3.38 and 4.49 µM, respectively. Notably, DEC‐1 showed a cytotoxicity profile nearly identical to that of the standard drug. Using GraphPad Prism software, the IC_50_ values were determined (Figure 8) [46]. After conducting the MTT assay, the absorbance data was entered into the software to generate dose–response curves. The nonlinear regression analysis function, specifically log(inhibitor) verus normalized response was employed to calculate the IC_50_ values for each compound. This method allowed for accurate determination of the concentration required to inhibit 50% of cell viability. The results were then compared to the standard drug, tamoxifen, to assess the relative cytotoxicity of the decapeptides.

## 3 Experimental Section

### 3.1 Coupling of 2‐CTC Resin with Fmoc‐Leu‐OH and Synthesis of 1a–c

The 2‐CTC resin (with a loading capacity of 1.0 mmol/g) was introduced into the peptide synthesizer. The resin was initially rinsed with 10 mL of dichloromethane (DCM) before draining, followed by the addition of 10 mL of DCM to the resin, and it was kept for stirring for about 60 min to allow swelling and subsequently drained. Next, Fmoc‐Leu‐OH (3.0 eq.) was dissolved in DCM and introduced into the reaction vessel. This was followed by the addition of DIPEA (6 eq.), and the mixture was allowed to stir at room temperature (25°C) for about 2 h. After the completion of the reaction, the peptidyl resin was filtered and rinsed twice with DCM and once with dimethylformamide (DMF).

A solution comprising DIPEA, methanol (MeOH), and DCM in a 1:2:8 ratio was utilized to cap any unreacted functional groups on the resin. An ultraviolet (UV) spectrophotometer was employed to monitor the loading percentage.

The deprotection of the Fmoc group was carried out by using a solution of 20% 10 mL piperidine/DMF. The reaction was monitored using the Kaiser test. The appearance of the blue color signified the completion of the deprotection process. The peptidyl resin was then washed with DMF (two times), isopropyl alcohol (IPA) (one time), and DMF (three times).

### 3.2 Synthesis of 2a–c

The SPPS Fmoc/t‐Bu (t‐Butyl)/Boc (t‐Butyloxycarbonyl) protocol was followed to synthesize the desired intermediates (2a–c). The Fmoc‐Leu‐2‐CTC resin was swollen in DMF (20 mL) for 30 min prior to the reaction. The intermediates were synthesized using a CSBio peptide synthesizer.

The synthesis of each intermediate involved the following protocols of coupling and deprotection. Fmoc amino acid (3.0 eq., or 3 mmol, referring to the initial resin loading), HOBt.H_2_O (3.0 eq., or 3 mmol), HBTU (3 eq., or 3 mmol), and DIPEA (6.0 eq., or 6 mmol) were dissolved in DMF (6 mL) for the coupling reaction. The coupling reactions were stirred for 60 min, and completion was confirmed using the Kaiser test (colorless test solution and beads). The peptidyl resin was filtered and washed with DMF (five times).

For deprotection of Fmoc group same protocol was followed as mentioned in the synthesis of 1a–c.

### 3.3 Synthesis of 3a–c

For side chain and CTC resin cleavage, a cleavage cocktail of TFA:TIS:H_2_O (80:10:10) in a volume of 10 mL per gram of the protected peptidyl resin was prepared. The protected peptidyl resin was allowed to stir for 3 h at 20°C–25°C in this cleavage cocktail. After the 3 h cleavage period, the reaction mixture was filtered, and the filtrate was precipitated with diisopropyl ether (DIPE) in a 50% volume ratio. The precipitated mixture was then filtered and washed with DIPE (three times) in a 10% volume ratio. The desired products were obtained by drying the wet cake under vacuum at 30°C.

### 3.4 Procedure for MTT Assay

The MTT assay (3‐[4,5‐dimethylthiazol‐2‐yl]‐2,5‐diphenyltetrazolium bromide) was employed to evaluate the anticancer potential of the synthesized compounds against the MCF‐7 human breast cancer cell line. MCF‐7 cells were seeded into a 96‐well plate at a density of 1 × 10^4^ cells per well and allowed to adhere for 24 h. Subsequently, the cells were treated with the test compounds at concentrations of 0.1, 1, 2, 5, and 10 µM, prepared in plain Dulbecco's Modified Eagle Medium (DMEM) media [47, 48, 49]. After 24 h of incubation, MTT solution (100 µg per well; Sigma‐Aldrich) was added, and the plates were incubated for an additional 3–4 h to allow the formation of formazan crystals. The resulting formazan was dissolved in DMSO, and absorbance was measured at 570 nm using a microplate reader. Tamoxifen served as the reference standard for comparison of cytotoxic activity [50, 51, 52].

### 3.5 Procedure for Molecular Docking Studies

In this study, three peptides were docked using molecular docking to investigate their potential interactions with the target protein. By simulating peptide‐receptor binding, the study identifies optimal conformations and predicts the strength and specificity of interactions. For molecular docking studies, the Glide module of the Schrodinger software was employed to investigate the affinity between the synthesized decapeptides and the targeted proteins to determine the potent decapeptide that has anticancer properties.

The targeted protein, Keap‐1 (Kelch‐like ECH‐associated protein 1) (PDB ID: 7Q6S) and XO (PDB ID: 2CKJ), were obtained from the Protein Data Bank. The protein was prepared by optimizing the crystal structure using Schrodinger's “Protein Preparation Wizard”. The optimization included removing water molecules, adding polar hydrogens and missing side chains, and eliminating the cocrystal ligand. Further optimization of the structure was done by applying the OPLS3 force field. The PDB IDs were selected such that they provide better resolution and have a cocrystal which can be employed for comparison. Also, the check was made to ensure that the selected IDs do not possess mutations.

The grid was generated by focusing on the cocrystal ligand of the targeted proteins by employing the GLIDE module. The grid is generated with the cocrystalized ligand in the center. The ligand optimization and preparation were done using Schrodinger's LigPrep module. The ligands are prepared generally at 7.4 pH, to generate the best‐fit conformer, per ligand. Standard Precision Docking mode was used for the docking study of the synthesized series of decapeptides [53, 54].

Peptides, unlike small molecules, possess multiple rotatable bonds and adopt various conformations due to backbone and side‐chain flexibility. In this study, we minimized conformational bias by performing energy minimization and conformational sampling of each peptide prior to docking using the OPLS4 force field in the LigPrep module. The most stable low‐energy conformers were then used for docking. This approach, while not exhaustive, provides a reasonable representation of peptide flexibility within the limits of rigid‐receptor docking. Glide SP uses a grid‐based search and rigid‐receptor approximation, which is computationally efficient but less exhaustive for large, flexible ligands. To improve sampling, we increased the number of initial poses generated per ligand and retained the top‐ranked poses based on GlideScore and visual inspection of hydrogen bonding, hydrophobic interactions, and backbone fit within the binding pocket. These settings improved sampling diversity while maintaining computational feasibility. Accurate protonation states are crucial for peptides because they affect charge distribution and binding affinity. The Epik module (Schrödinger Suite) was used to assign protonation states at physiological pH (7.4), and the N‐ and C‐termini of peptides were capped (acetylated and amidated) to mimic their natural electronic environment and reduce artificial end‐charge effects that can distort docking results. We recognize that Glide SP employs a rigid‐receptor model and does not account for receptor flexibility upon ligand binding. To address this, we focused on receptor structures cocrystallized with ligands (Keap‐1, PDB ID: 7Q6S; XO, PDB ID: 2CKJ), ensuring that active site residues were already in conformations conducive to ligand accommodation. While molecular dynamics (MD) simulations were beyond the current study's scope, molecular dynamic‐based refinement would allow dynamic relaxation of peptide–protein complexes and provide more reliable estimates of binding stability and interaction persistence over time. We plan to perform such simulations in our ongoing work.

## 4 Conclusion

The finding of the study provided the significant stride in the development of cancer‐based therapeutics by providing the potent anticancer agents, which can be taken forward for the advanced research studies to unveil its potential as a therapeutic. The synthesis of decapeptides and their subsequent assessment was done by using the MCF‐7 breast cancer cell line, which showed that DEC‐1 has significant inhibition of cell proliferation and superior efficacy compared to the other peptides. Overall, the data indicates that DEC‐1 exhibits cytotoxicity nearly equivalent to that of tamoxifen, the reason behind it can be the presence of 1,2,3,4‐tetrahydroisoquinoline‐3‐carboxylic acid (Tic acid), as it indirectly affects the signaling pathways for estrogen receptors and reduces the estrogen levels, which can inhibit the tumor growth. Also, tamoxifen directly blocks estrogen receptor; therefore after a certain dosage, resistance can occur due to alteration or mutation in signaling pathways. This can be overriden by DEC‐1 due to its nature, multiple receptor interaction, and stability. However detailed study is required to understand the mechanism of action and in vivo efficacy of DEC‐1 by preclinical trials.

The in silico approach was employed to elucidate the interactions of DEC‐1 with two critical cancer‐related targets: XO and Kelch‐like ECH‐associated protein 1 (Keap‐1). The computational analysis revealed strong binding affinities and stable interactions with these targets, suggesting potential pathways through which DEC‐1 might exert its effects. In conclusion, the results from both biological and computational studies suggest that DEC‐1 can be a potent molecule for anticancer treatment, though further research is required to add this molecule in the pipeline for cancer therapeutics.

## Supporting Information

Additional supporting information can be found online in the Supporting Information Section. **Supporting Fig. S1:** Structure of decapeptides 3a‐c. 3a) His‐Gly‐Arg‐His‐Lys‐Tic‐Glu‐Ser‐Trp‐Leu 3b) Arg‐Cys‐Lys‐Arg‐His‐Tic‐Glu‐Ser‐Trp‐Leu 3c) Lys‐Cys‐Arg‐Lys‐His‐Tic‐Glu‐Ser‐Trp‐Leu. **Supporting Fig. S2:** Synthetic route of peptide **DEC‐1**. **Supporting Fig. S3:** Synthetic route of peptide **DEC‐2**. **Supporting Fig. S4:** Synthetic route of peptide **DEC‐3**. **Supporting Fig. S5:** Structural representation of DEC‐1. **Supporting Fig. 6**: HPLC chromatogram of DEC‐1 showing purity profile at 215 nm. **Supporting Fig. S7:** ^1^H NMR Spectrum of DEC‐1. **Supporting Fig. S8:** ^13^C NMR data of DEC‐1. **Supporting Fig. S9:** Mass Spectroscopy data of DEC‐1 showing M+1 peak. (Molecular Wt: 1308.47 gmol^−1^). **Supporting Fig. S10:** Structural representation of DEC‐2. **Supporting Fig. S11:** HPLC chromatogram of DEC‐2 showing purity profile at 215 nm. **Supporting Fig. S12:** ^1^H NMR data of DEC‐2. **Supporting Fig. S13:** ^13^C NMR data of DEC‐2. **Supporting Fig. S14:** Mass Spectroscopy data of DEC‐2 showing M/2 peak. (Molecular wt: 1373.60 gmol^−1^. **Supporting Fig. S15:** Structural representation of DEC‐3. **Supporting Fig. S16:** HPLC chromatogram of DEC‐3 showing purity profile at 215 nm. **Supporting Fig. S17:** ^1^H NMR data of DEC‐3. **Supporting Fig. S18:** ^13^C NMR data of DEC‐3. **Supporting Fig. S19:** Mass Spectroscopy data of DEC‐3 showing M‐1 peak. (Molecular Wt: 1345.59 gmol^−1^. **Supporting Fig. S20:** 2D interactions of reference molecule (91M) with Keap‐1 (7Q6S). **Supporting Fig. S21:** 2D interactions of DEC‐1 with Keap‐1 (7Q6S). **Supporting Fig. S22:** 2D interactions of DEC‐2 with Keap‐1 (7Q6S). **Supporting Fig. S23:** 2D interactions of DEC‐3 with Keap‐1 (7Q6S). **Supporting Fig. S24:** 2D interactions of reference molecule (FAD) with Xanthine Oxidase (2CKJ). **Supporting Fig. S25:** 2D interactions of DEC‐1 with Xanthine Oxidase (2CKJ). **Supporting Fig. S26:** 2D interactions of DEC‐2 with Xanthine Oxidase (2CKJ). **Supporting Fig. S27:** 2D interactions of DEC‐3 with Xanthine Oxidase (2CKJ).

## Conflicts of Interest

The authors declare no conflicts of interest.

## Supporting information

Supplementary Material

## Data Availability

Route of synthesis, spectral data, and molecular docking study of all newly synthesized compounds is given in supplementary information file.
