# Supplementary material for: Synthesis of 1,2,3,4‐Tetrahydroisoquinoline‐3‐Carboxylic Acid‐Embedded Decapeptides: In Silico Studies and In Vitro Evaluation Against Breast Cancer
Source: ChemistryOpen. 2025 Dec 17;15(4):e202500282. doi: 10.1002/open.202500282 (PMC13052075; doi:10.1002/open.202500282)
Supplement: Supplementary file 1 — Supplementary Material [file OPEN-15-e202500282-s001.pdf]

Synthesis of 1,2,3,4-Tetrahydroisoquinoline-3-Carboxylic Acid-Embedded Decapeptides: *In Silico* studies and *In Vitro* Evaluation Against Breast Cancer

Sunil R. Tivari[a$], Naurin Lalani[a$], Pritam Bagwe[b], Prashant K. Chandole[a], Ramesh Pawar[a], Enrique Delgado-Alvarado[c], Abdallah Othman Ghnaim[d], Yashwantsinh Jadeja[a*]

[a]Department of Chemistry, Marwadi University, Rajkot-360003, Gujarat, India.

[b] Department of Pharmaceutical Sciences and Technology, Institute of Chemical Technology, Mumbai

[c]Micro and Nanotechnology Research Center, Universidad Veracruzana, Blvd. Av. Ruiz Cortines No. 455 Fracc. Costa Verde, Boca del Río 94294, Mexico.

[d]Department of Chemistry, Faculty of Science, Al-Balqa' Applied University-Salt, Jordan

*Corresponding Author: Dr. Yashwantsinh Jadeja,
Associate Professor,
Department of Chemistry, Faculty of Science,
Marwadi University, Rajkot-360003, Gujarat, India
+919925525586
drysjadeja@gmail.com
yashwantsinh.jadeja@marwadieducation.edu.in

$ Both authors have equal contributions.

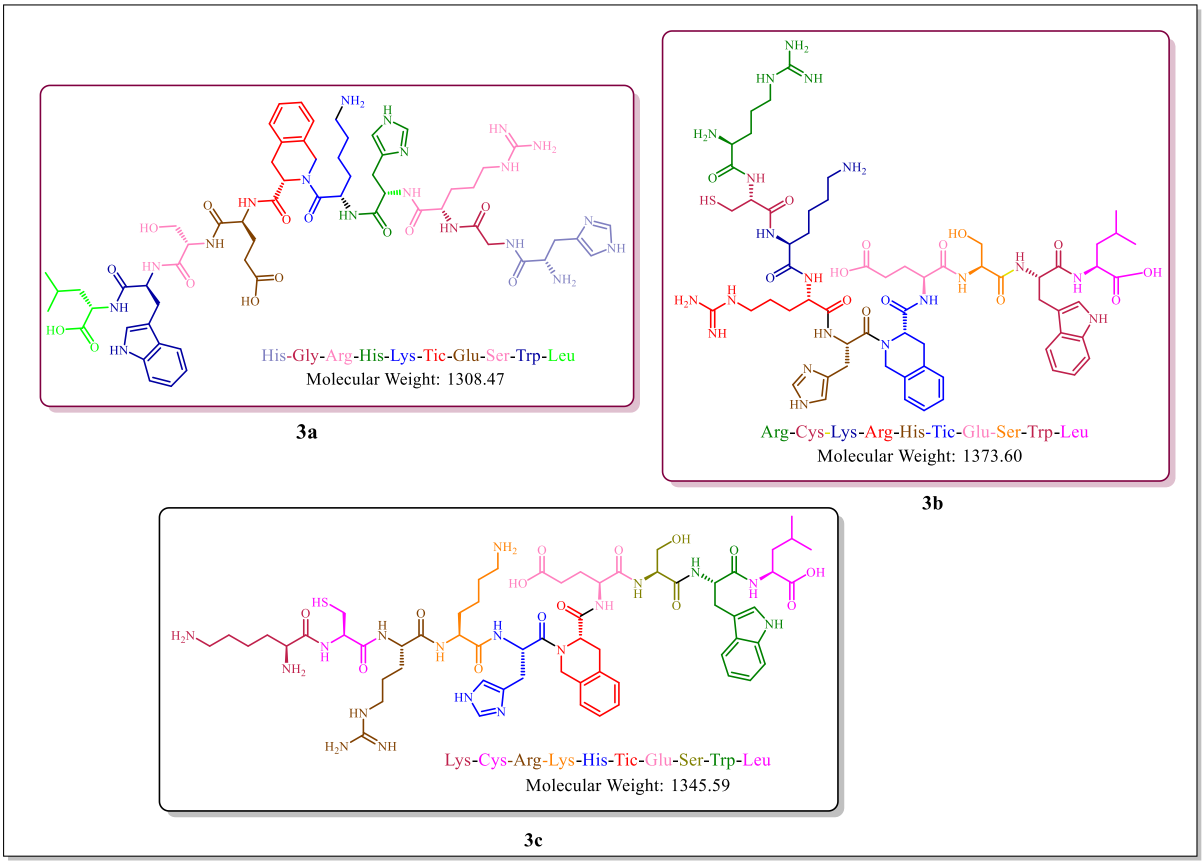

Figure 1. Structure of decapeptides 3a-c. 3a) His-Gly-Arg-His-Lys-Tic-Glu-Ser-Trp-Leu 3b) Arg-Cys-Lys-Arg-His-Tic-Glu-Ser-Trp-Leu 3c) Lys-Cys-Arg-Lys-His-Tic-Glu-Ser-Trp-Leu

Material and Methods

The synthesis of decapeptides (Figure 1) was carried out by employing 2-chlorotrityl chloride (2-CTC) resin, which exhibited excellent loading efficiency. This resin is commonly employed for synthesizing low molecular weight peptides and was acquired from Merck. The process involved the use of Fmoc (9-fluorenylmethoxycarbonyl)-protected L-amino acids, procured from Sichuan, China. Triisopropylsilane (TIS), and HBTU (2-(1H-benzotriazol-1-yl)-1,1,3,3-tetramethyluronium) were obtained from survival chemical. phenol was acquired from SD fine chemicals, while diisopropylethylamine (DIPEA), HOBt.H2O (N-hydroxybenzotriazole monohydrate), and trifluoroacetic acid (TFA) were sourced from Spectrochem. The cell line (MCF-7 breast cancer) for MTT assay was purchased from Merck.

A fully automated CSBio peptide synthesizer (CS136X) was employed for synthesis. The melting point was determined using open capillary method. The progress of the reaction was monitored using kaiser test. Following synthesis, the peptides were purified through the use of ethyl acetate and n-hexanes in combination.

For characterization, Mass spectra were obtained using a Shimadzu LC-MS. ^{1}H and ^{13}C were obtained using Bruker Avance 400 MHz NMR spectrophotometer was employed to record the NMR spectra in DMSO-d_6 solvent.

Route of synthesis of Amphipathic cationic peptides 3a-c

DEC-1:

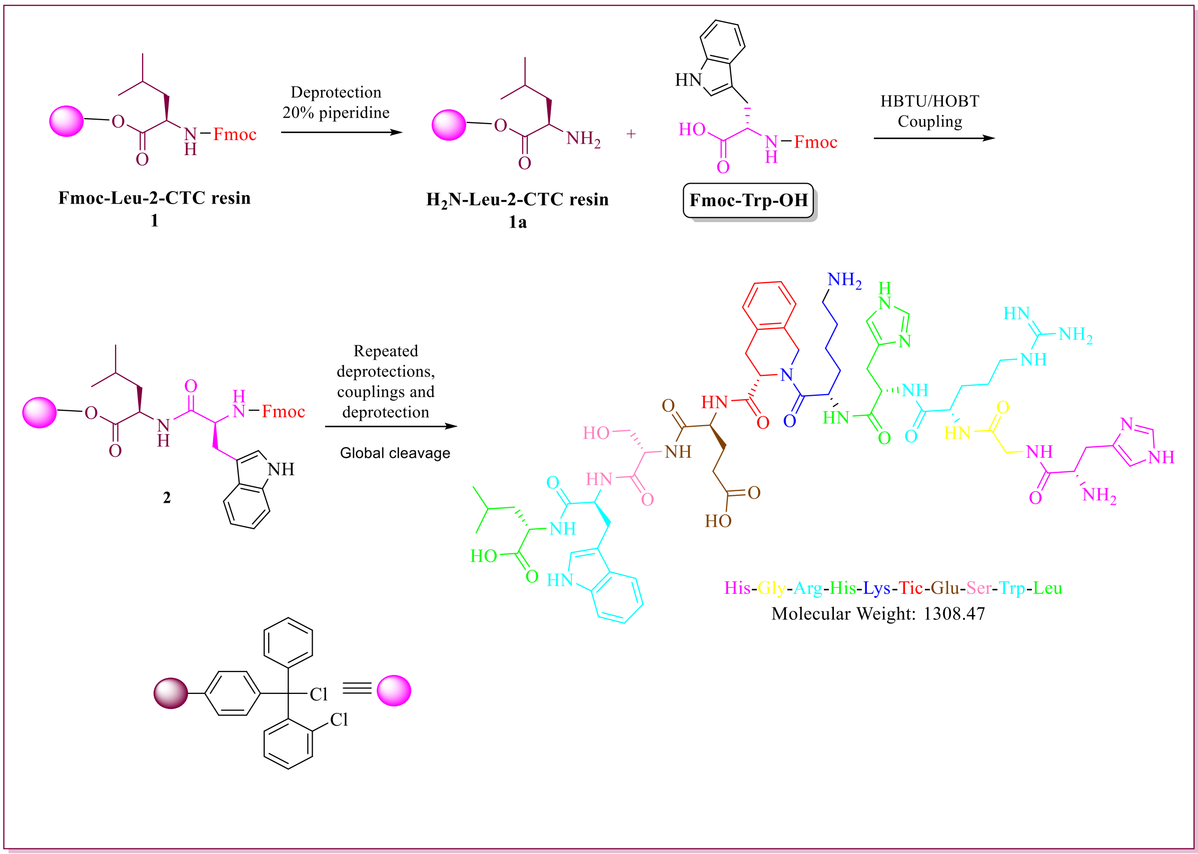

Figure 2. Synthetic route of peptide **DEC-1**

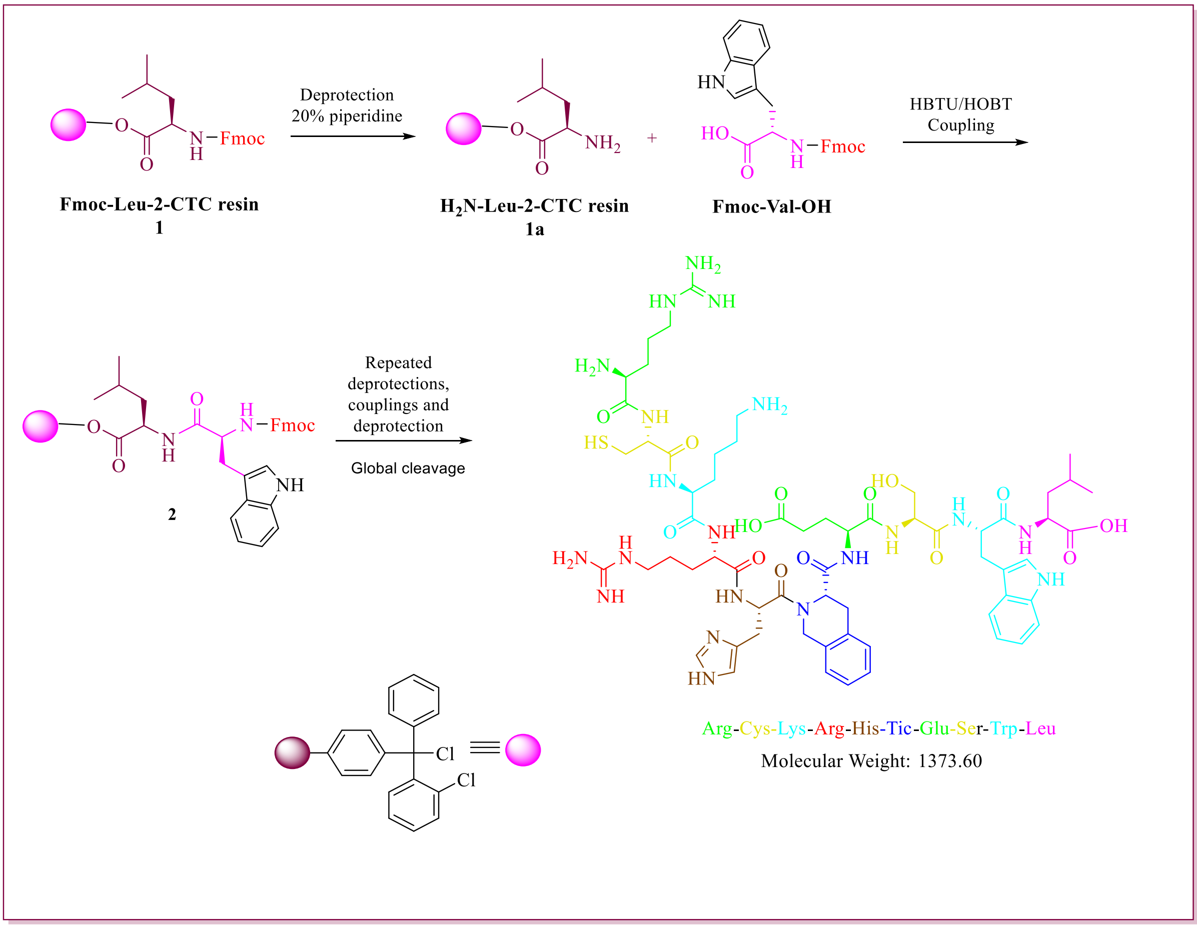

Figure 3. Synthetic route of peptide **DEC-2**

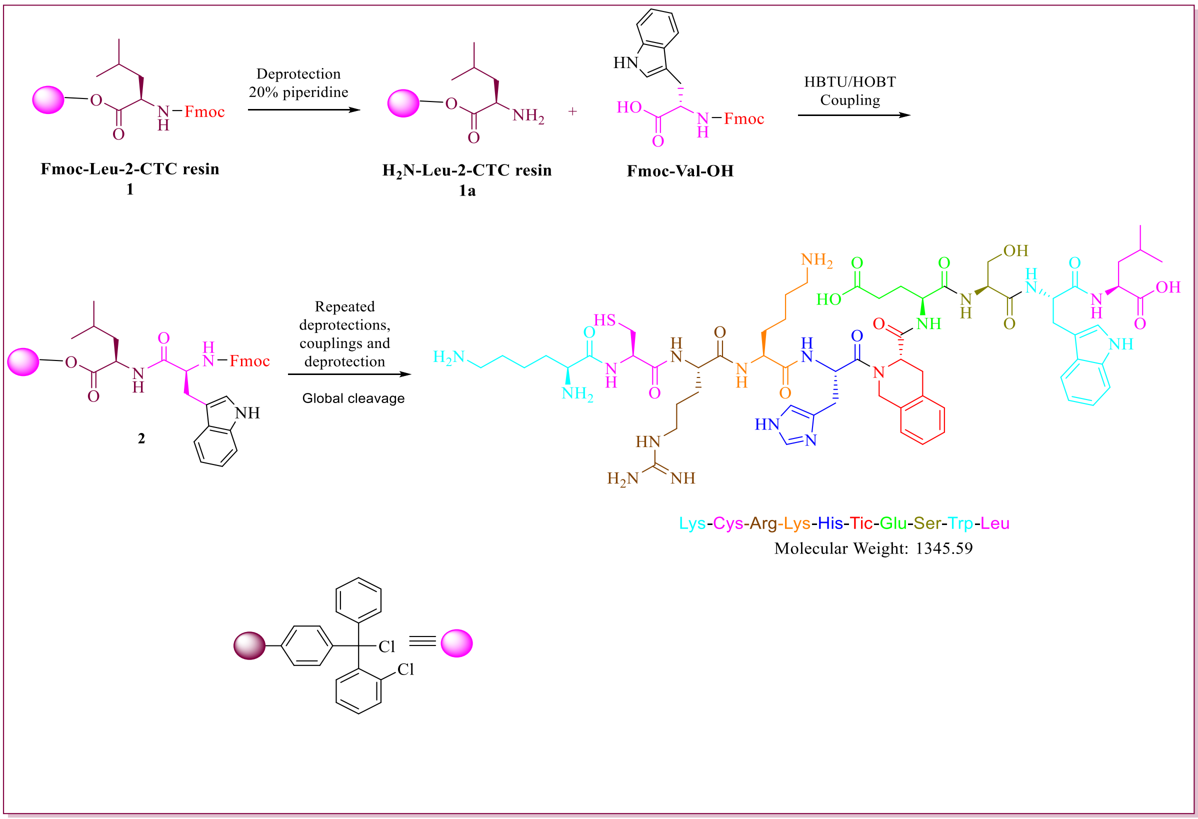

Figure 4. Synthetic route of peptide **DEC-3**

Characterization Data:

DEC-1:

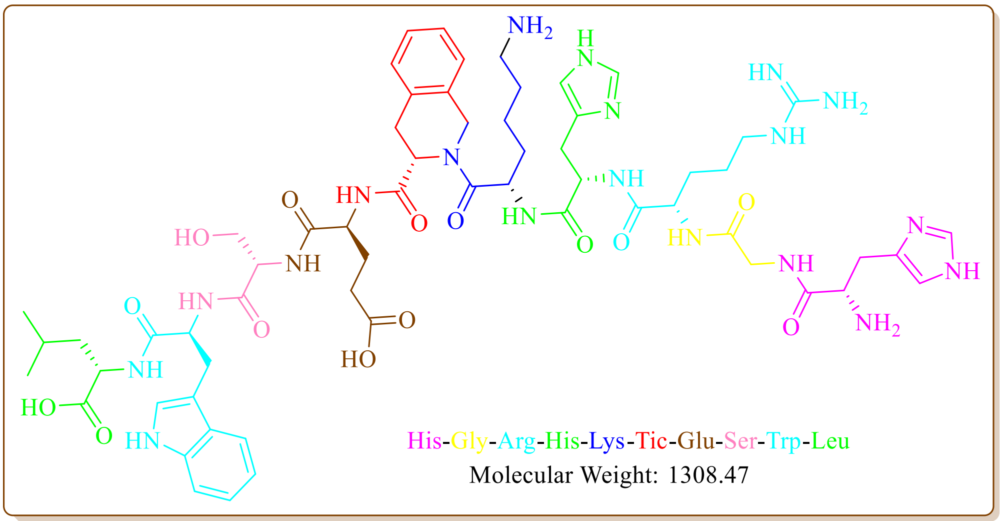

Figure 5. Structural representation of DEC-1

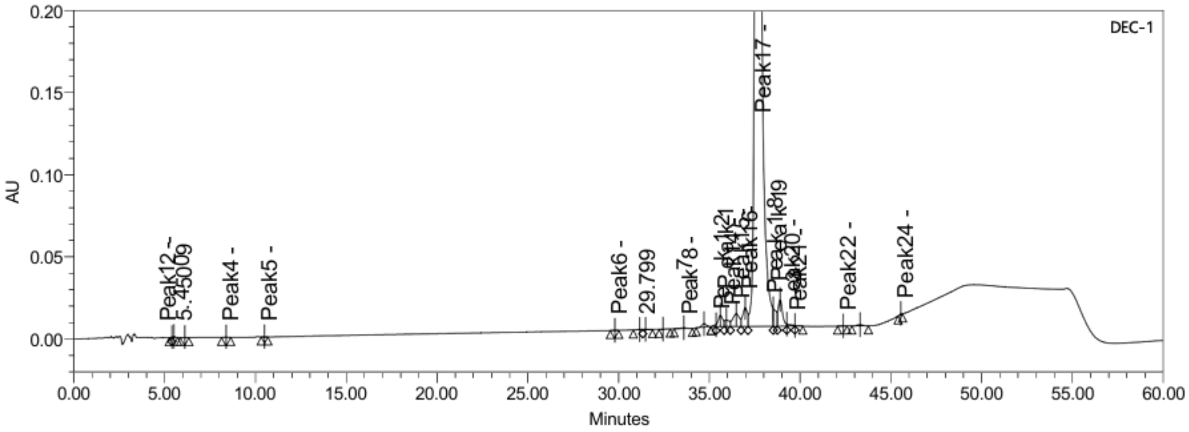

Figure 6. HPLC chromatogram of DEC-1 showing purity profile at 215nm.

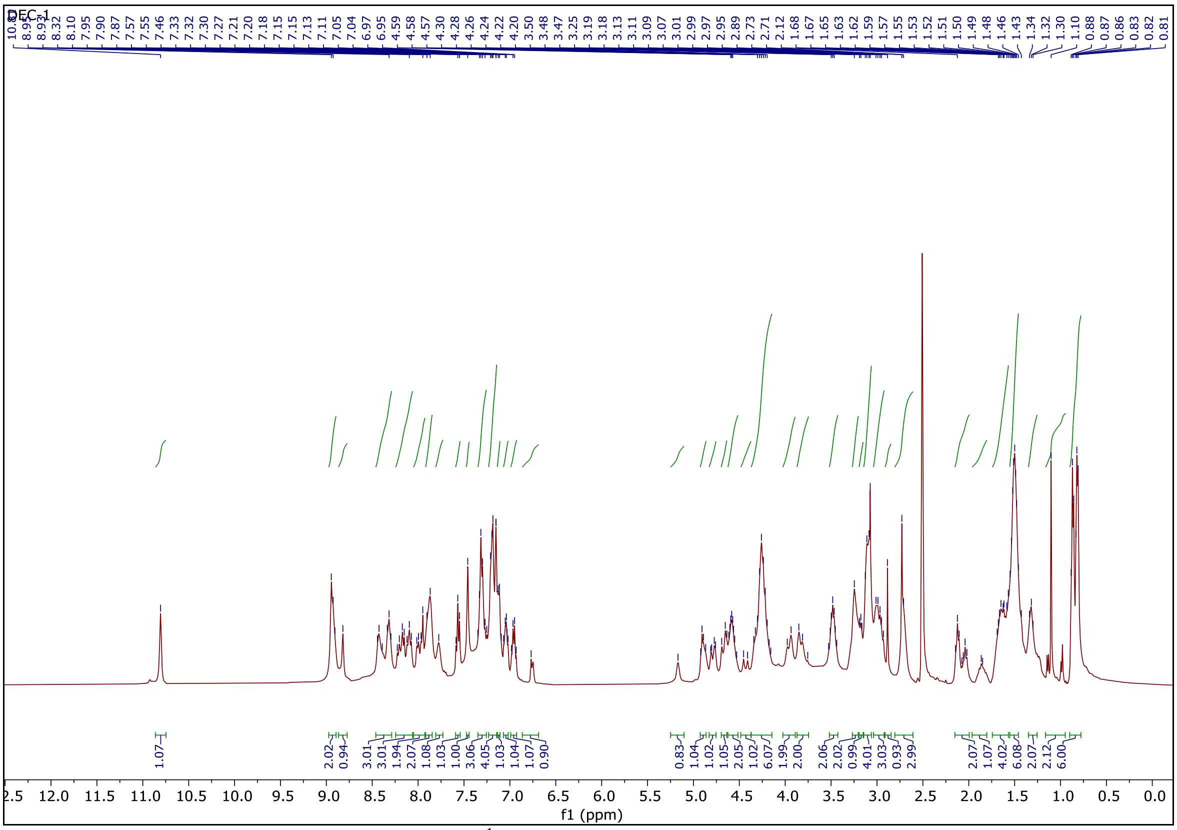

Figure 7. ^{1}H NMR Spectrum of DEC-1

Yield: 90%,

Melting Point: 198-203 ºC

% Purity in HPLC: 93.83%

^{1}**H NMR** (400 MHz, DMSO-d_6) δ 10.81 (s, 1H), 8.94 (d, J = 8.1 Hz, 2H), 8.82 (s, 1H), 8.37 (dt, J = 37.6, 7.4 Hz, 3H), 8.14 (dt, J = 31.2, 11.0 Hz, 3H), 8.05 – 7.93 (m, 2H), 7.89 (d, J = 14.5 Hz, 2H), 7.77 (s, 1H), 7.57 (t, J = 7.6 Hz, 1H), 7.46 (s, 1H), 7.32 (t, J = 6.9 Hz, 3H), 7.23 – 7.14 (m, 4H), 7.12 (d, J = 5.5 Hz, 1H), 7.07 – 7.02 (m, 1H), 6.96 (q, J = 6.8 Hz, 1H), 6.77 (s, 1H), 5.17 (s, 1H), 4.90 (q, J = 9.6, 7.7 Hz, 1H), 4.79 (dd, J = 16.4, 5.0 Hz, 1H), 4.67 (d, J = 15.6 Hz, 1H), 4.58 (tq, J = 13.5, 8.7, 6.6 Hz, 2H), 4.43 (d, J = 17.1 Hz, 1H), 4.27 (ddt, J = 25.2, 16.7, 7.6 Hz, 6H), 3.96 (d, J = 16.6 Hz, 2H), 3.83 (d, J = 16.2 Hz, 2H), 3.47 (dt, J = 10.9, 6.3 Hz, 2H), 3.25 (s, 2H), 3.17 (d, J = 4.7 Hz, 1H), 3.10 (dd, J = 15.6, 6.1 Hz, 4H), 3.04 – 2.92 (m, 3H), 2.89 (s, 1H), 2.72 (d, J = 7.7 Hz, 3H), 2.08 (dt, J = 33.5, 8.0 Hz, 2H), 1.86 (d, J = 6.0 Hz, 1H), 1.64 (tt, J = 22.5, 11.9 Hz, 4H), 1.55 – 1.46 (m, 6H), 1.35 – 1.25 (m, 2H), 1.10 (s, 2H), 0.85 (dt, J = 19.4, 5.7 Hz, 6H).

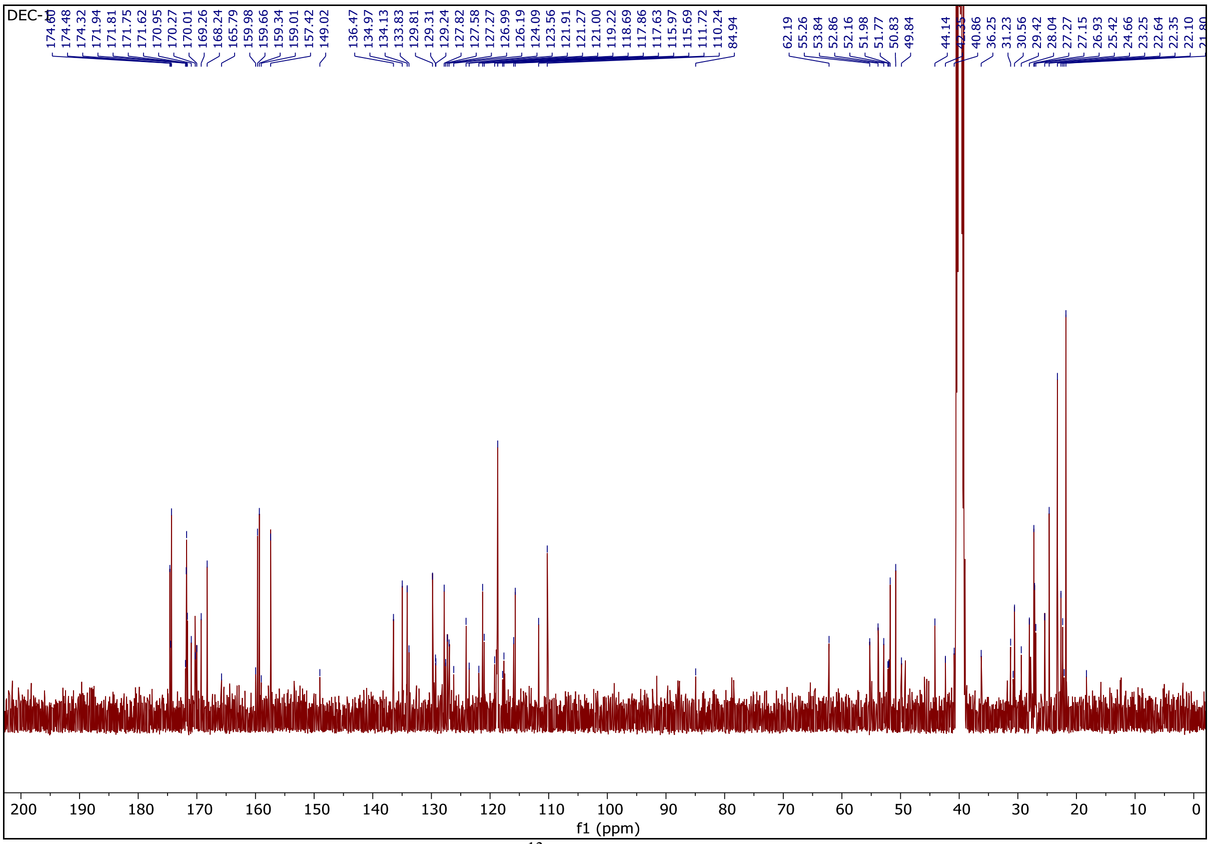

Figure 8. ^{13}C NMR data of DEC-1

^{13}C NMR (101 MHz, DMSO-d_6) δ 174.60, 174.48, 174.32, 171.81, 171.75, 171.62, 170.95, 170.27, 170.01, 169.26, 168.24, 159.98, 159.66, 159.34, 157.42, 136.47, 134.97, 134.13, 133.83, 129.81, 127.82, 127.58, 127.27, 126.99, 126.19, 124.09, 121.27, 118.69, 117.63, 115.97, 115.69, 111.72, 110.24, 62.19, 55.26, 53.84, 52.86, 52.16, 51.98, 51.77, 50.83, 44.14, 42.35, 30.79, 30.56, 29.42, 28.04, 27.27, 27.15, 26.93, 25.42, 24.66, 23.25, 22.64, 22.35, 21.80.

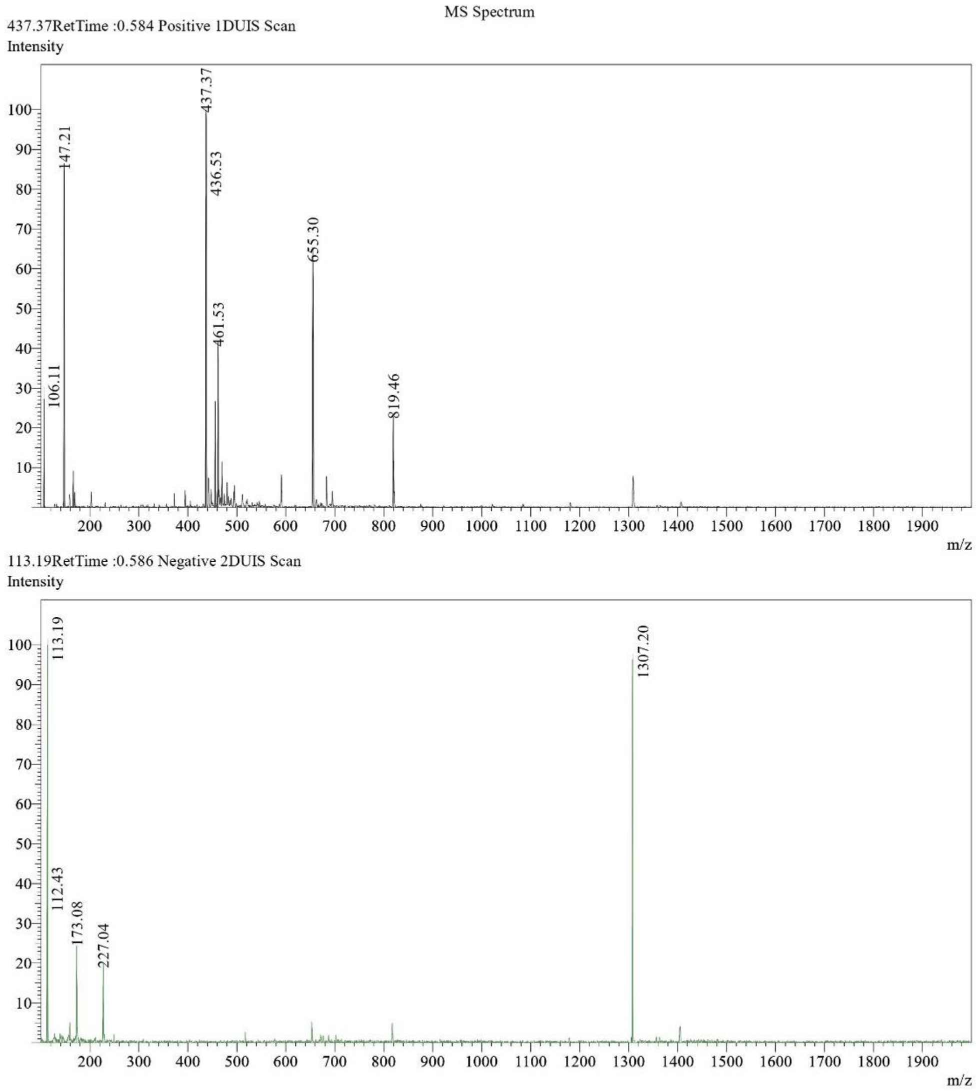

Figure 9. Mass Spectroscopy data of DEC-1 showing M+1 peak. (Molecular Wt: 1308.47gmol^{-1})

DEC-2:

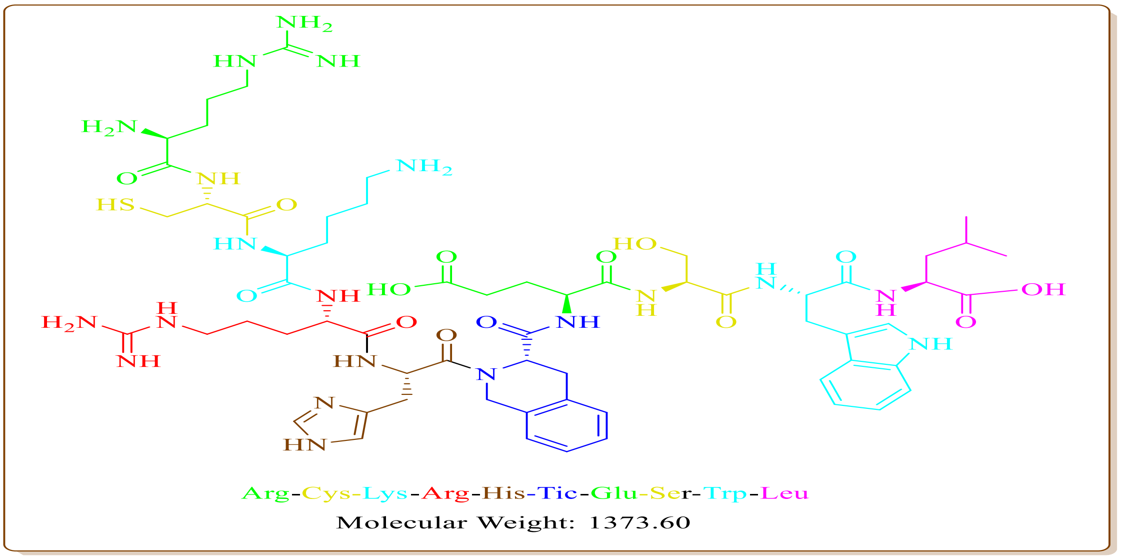

Figure 10. Structural representation of DEC-2

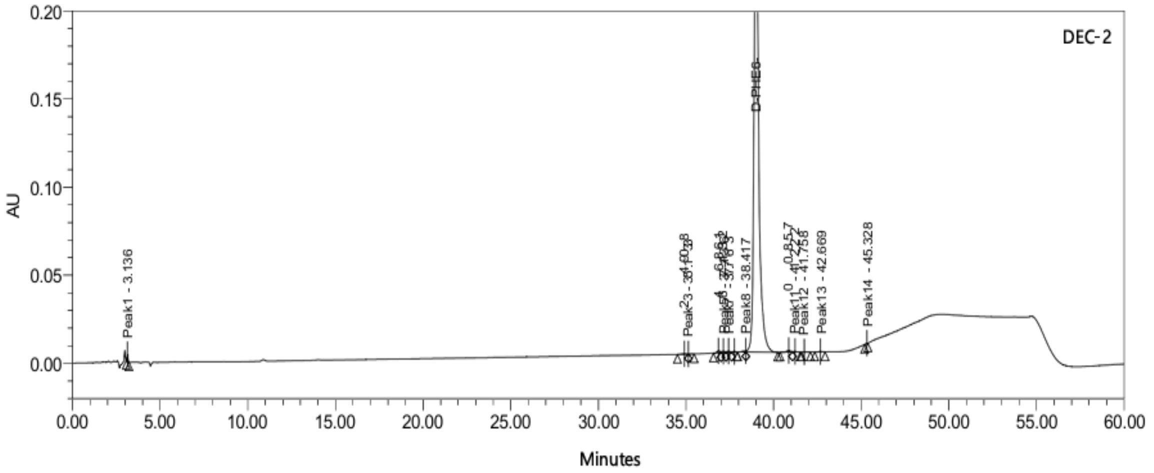

Figure 11: HPLC chromatogram of DEC-2 showing purity profile at 215nm.

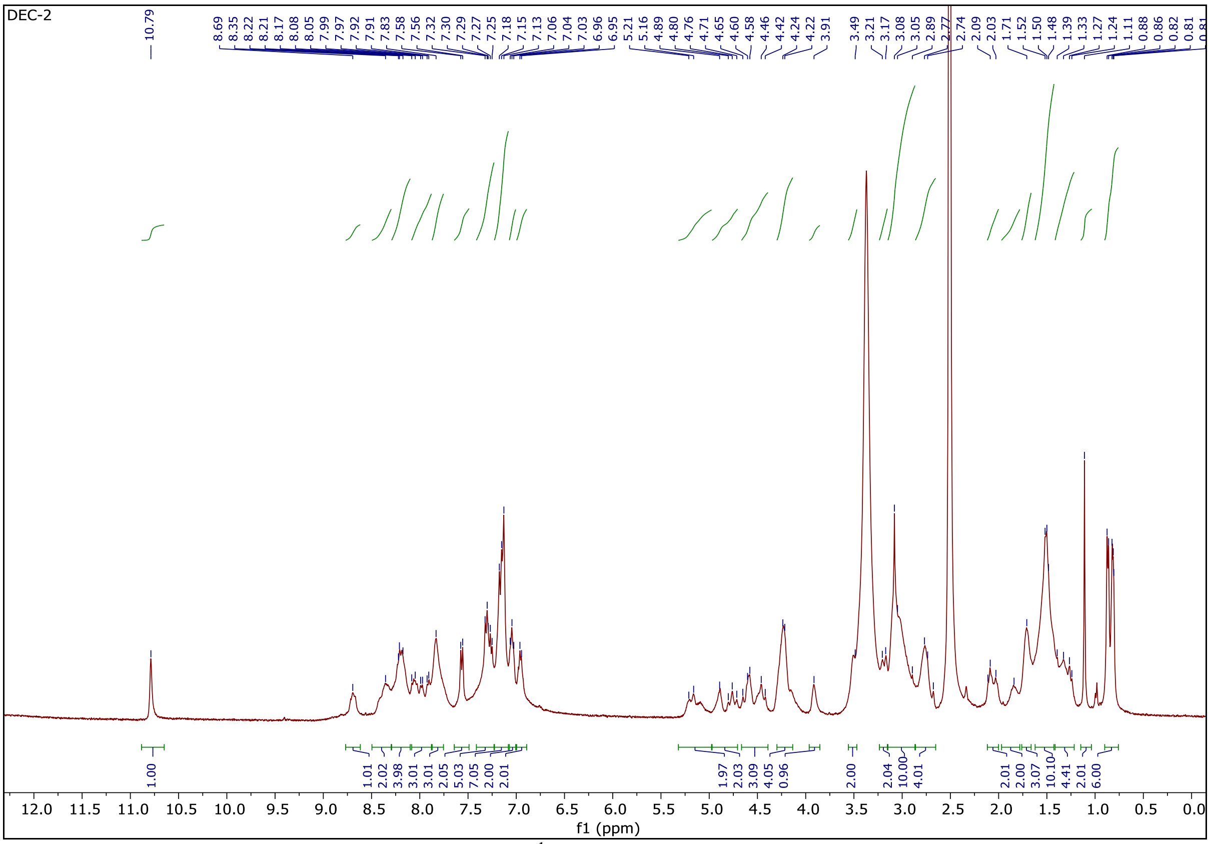

Figure 12. ^{1}H NMR data of DEC-2

Yield: 89%

Melting point: 203-205 ºC

% Purity in HPLC: 97.99%

^{1}**H NMR** (400 MHz, DMSO-d_6) δ 10.79 (s, 1H), 8.69 (s, 1H), 8.35 (s, 2H), 8.29 – 8.10 (m, 4H), 8.08 – 7.88 (m, 3H), 7.83 (s, 3H), 7.57 (d, J = 7.9 Hz, 2H), 7.41 – 7.23 (m, 5H), 7.23 – 7.08 (m, 7H), 7.04 (t, J = 7.4 Hz, 2H), 6.95 (d, J = 6.8 Hz, 2H), 5.19 (d, J = 20.0 Hz, 2H), 4.97 – 4.71 (m, 2H), 4.67 – 4.39 (m, 3H), 4.24 (s, 4H), 3.91 (s, 1H), 3.49 (s, 2H), 3.19 (d, J = 14.1 Hz, 2H), 3.15 – 2.87 (m, 10H), 2.75 (d, J = 13.1 Hz, 4H), 2.06 (d, J = 23.8 Hz, 2H), 1.84 (s, 2H), 1.71 (s, 3H), 1.62 – 1.43 (m, 10H), 1.41 – 1.22 (m, 4H), 1.11 (s, 2H), 0.90 – 0.76 (m, 6H).

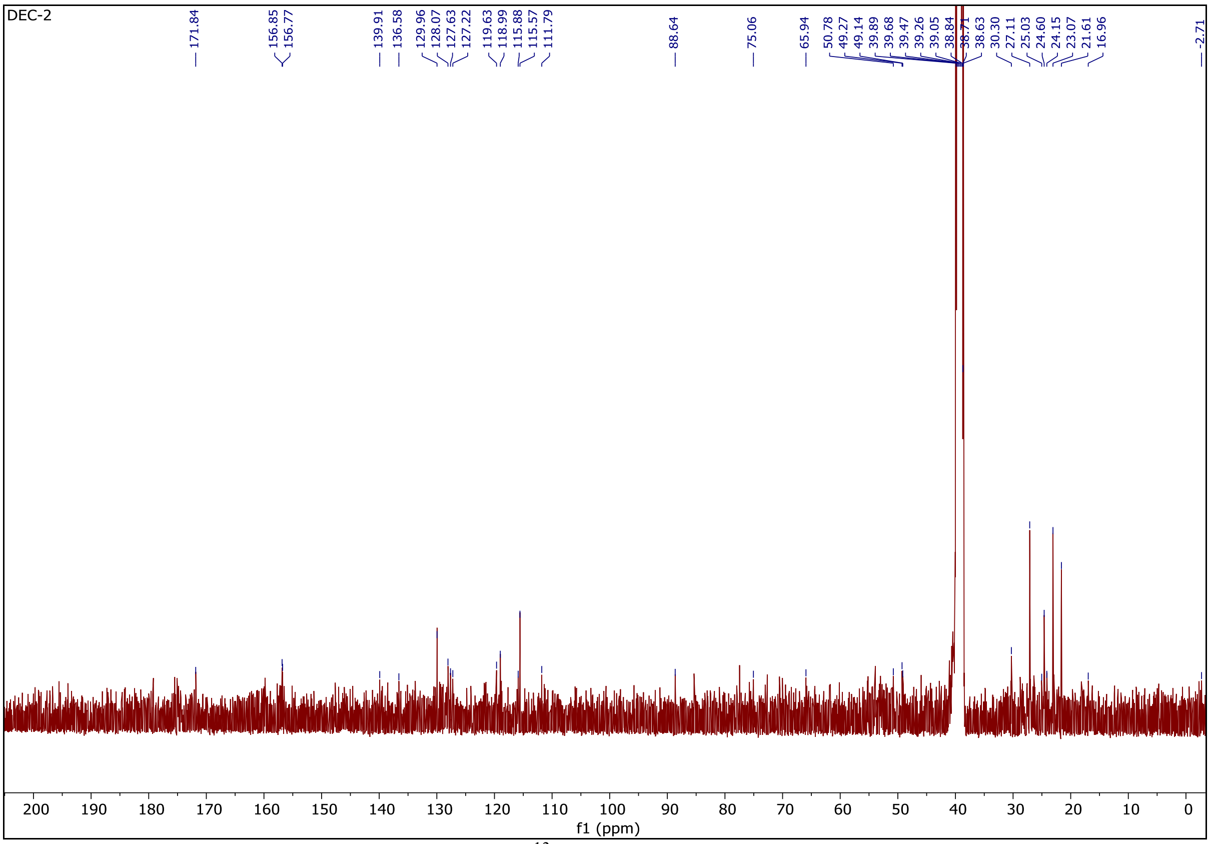

Figure 13. ^{13}C NMR data of DEC-2

^{13}C NMR (101 MHz, DMSO-d_6) δ 171.84, 156.85, 136.58, 129.96, 128.07, 127.63, 127.22, 119.63, 118.99, 115.57, 88.64, 75.06, 65.94, 50.78, 49.27, 30.30, 27.11, 25.03, 24.60, 24.15, 23.07, 21.61.

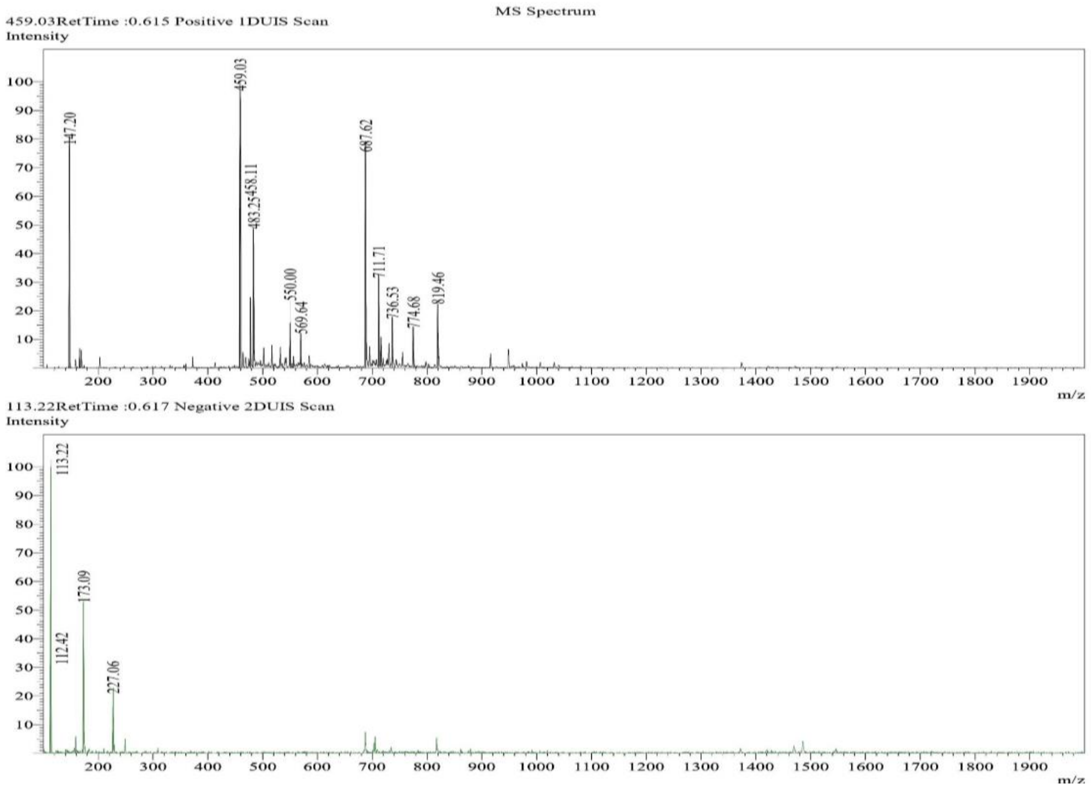

Figure 14. Mass Spectroscopy data of DEC-2 showing M/2 peak. (Molecular wt: 1373.60gmol^{-1}.

DEC-3:

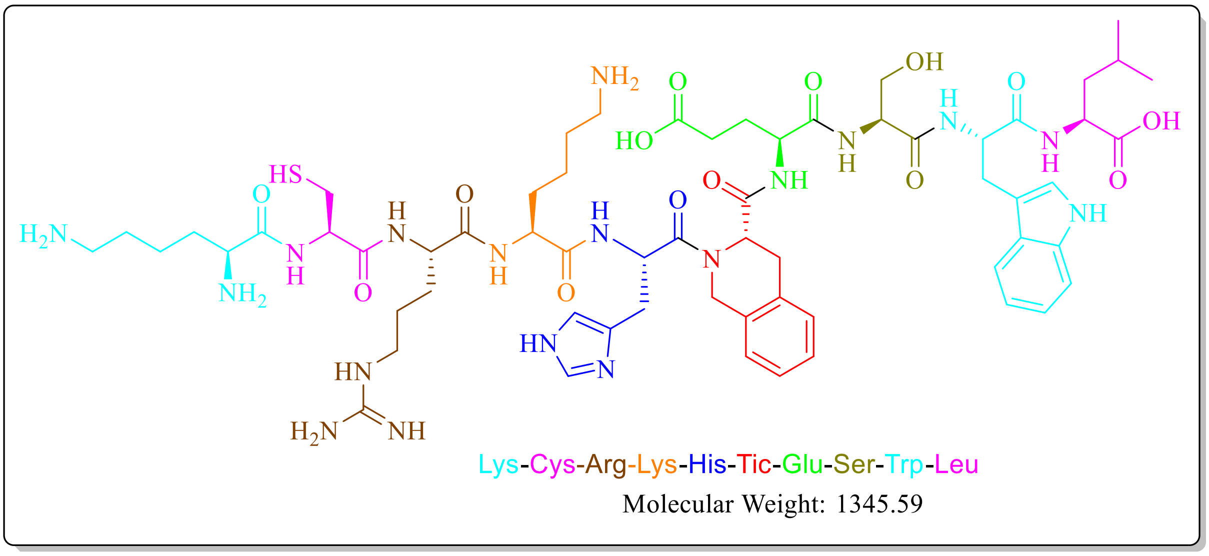

Figure 15. Structural representation of DEC-3

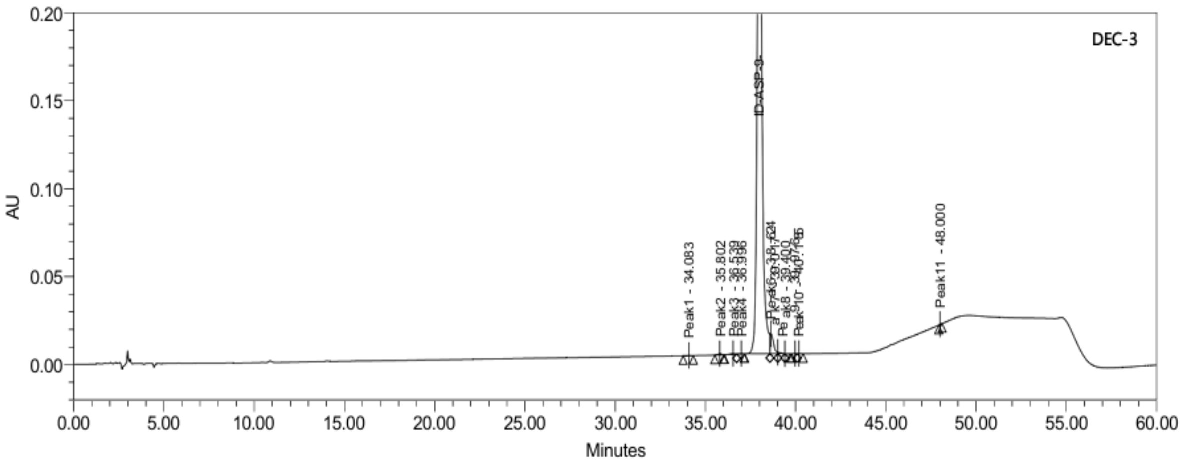

Figure 16: HPLC chromatogram of DEC-3 showing purity profile at 215nm.

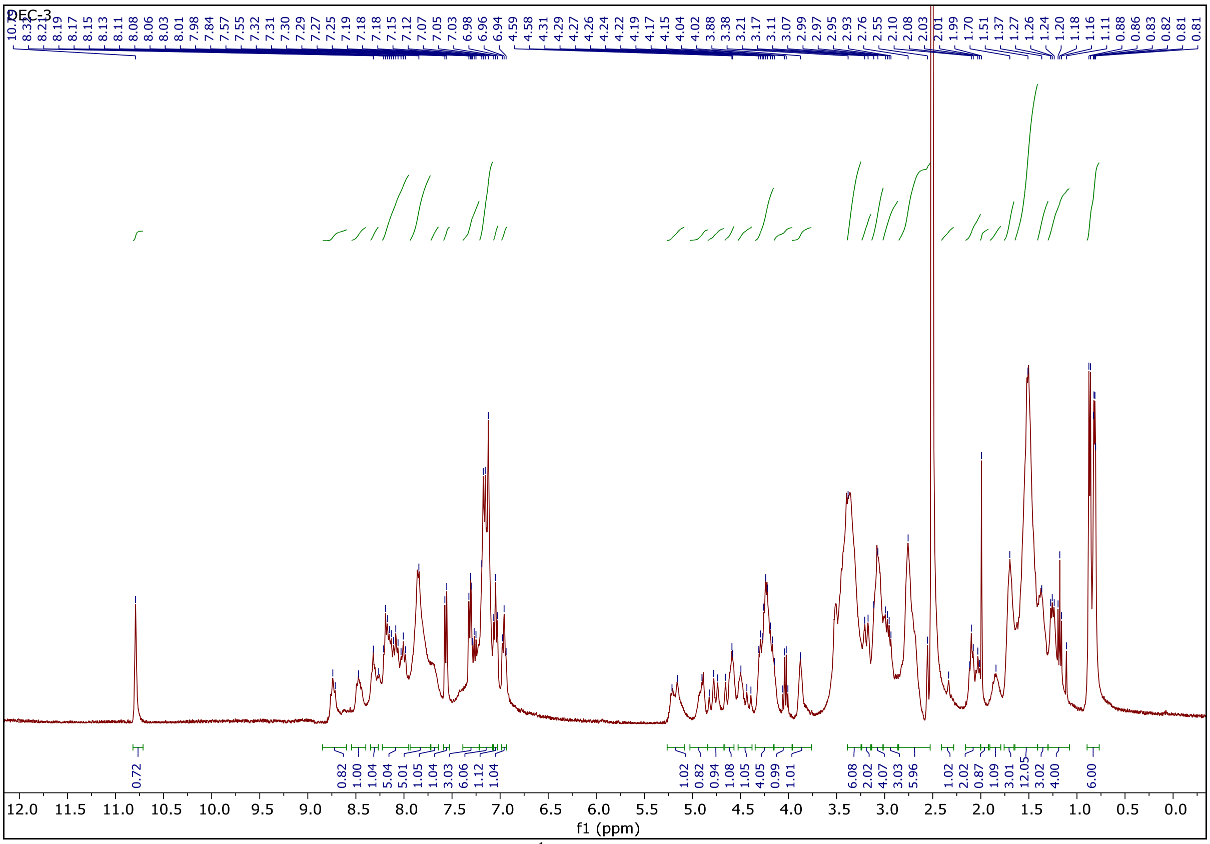

Figure 17. ^{1}H NMR data of DEC-3

Yield: 92%

Melting point: 199-202 ºC

% Purity in HPLC: 97.17%

^{1}**H NMR** (400 MHz, DMSO-d_6) δppm 10.79 (s, 1H), 8.73 (d, J = 9.9 Hz, 1H), 8.47 (s, 1H), 8.32 (s, 1H), 8.22 – 7.95 (m, 5H), 7.84 (s, 5H), 7.56 (d, J = 7.9 Hz, 1H), 7.39 – 7.22 (m, 3H), 7.21 – 7.08 (m, 6H), 7.05 (t, J = 7.5 Hz, 1H), 6.98 – 6.93 (m, 1H), 5.18 (d, J = 22.8 Hz, 1H), 4.90 (s, 1H), 4.84 – 4.67 (m, 1H), 4.66 – 4.57 (m, 1H), 4.52 – 4.38 (m, 1H), 4.24 (ddt, J = 28.1, 20.8, 7.7 Hz, 4H), 4.15 – 3.96 (m, 1H), 3.88 (s, 1H), 3.38 (s, 6H), 3.19 (d, J = 14.0 Hz, 2H), 3.07 (s, 4H), 2.96 (dd, J = 15.2, 8.2 Hz, 3H), 2.66 (d, J = 80.6 Hz, 6H), 2.34 (s, 1H), 2.06 (dd, J = 26.8, 7.8 Hz, 2H), 1.99 (s, 1H), 1.84 (s, 1H), 1.70 (s, 3H), 1.51 (s, 12H), 1.37 (s, 3H), 1.30 – 1.08 (m, 4H), 0.89 – 0.77 (m, 6H).

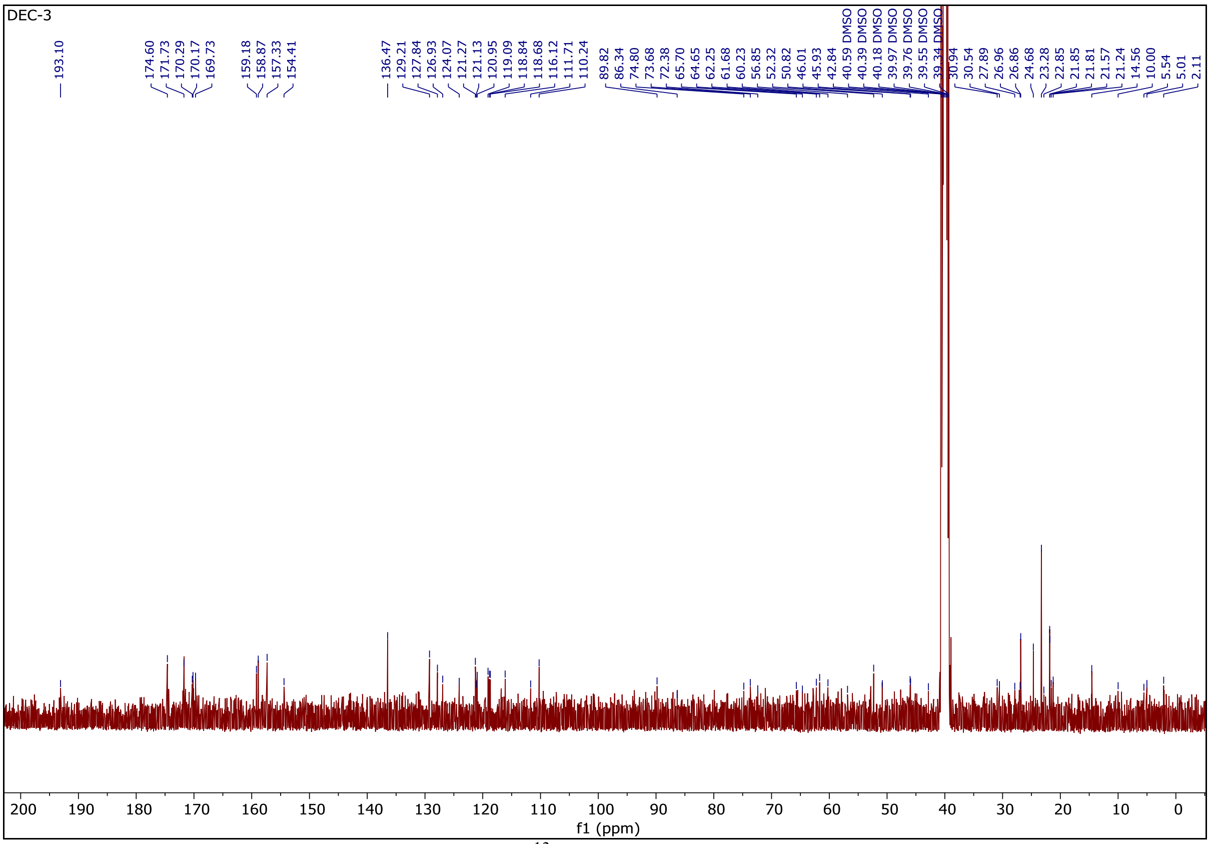

Figure 18. ^{13}C NMR data of DEC-3

^{13}C NMR (101 MHz, DMSO-d_6) δ 193.10, 174.60, 171.73, 170.29, 170.17, 169.73, 159.18, 158.87, 157.33, 154.41, 136.47, 129.21, 127.84, 126.93, 124.07, 121.27, 121.13, 120.95, 119.09, 118.84, 118.68, 116.12, 111.71, 110.24, 89.82, 86.34, 74.80, 73.68, 72.38, 64.65, 62.25, 61.68, 60.23, 56.85, 52.32, 50.82, 46.01, 45.93, 42.84, 30.94, 30.54, 27.89, 26.96, 26.86, 24.68, 23.28, 22.85, 21.85, 21.81, 21.57, 21.24, 14.56, 10.00, 5.54, 5.01, 2.11.

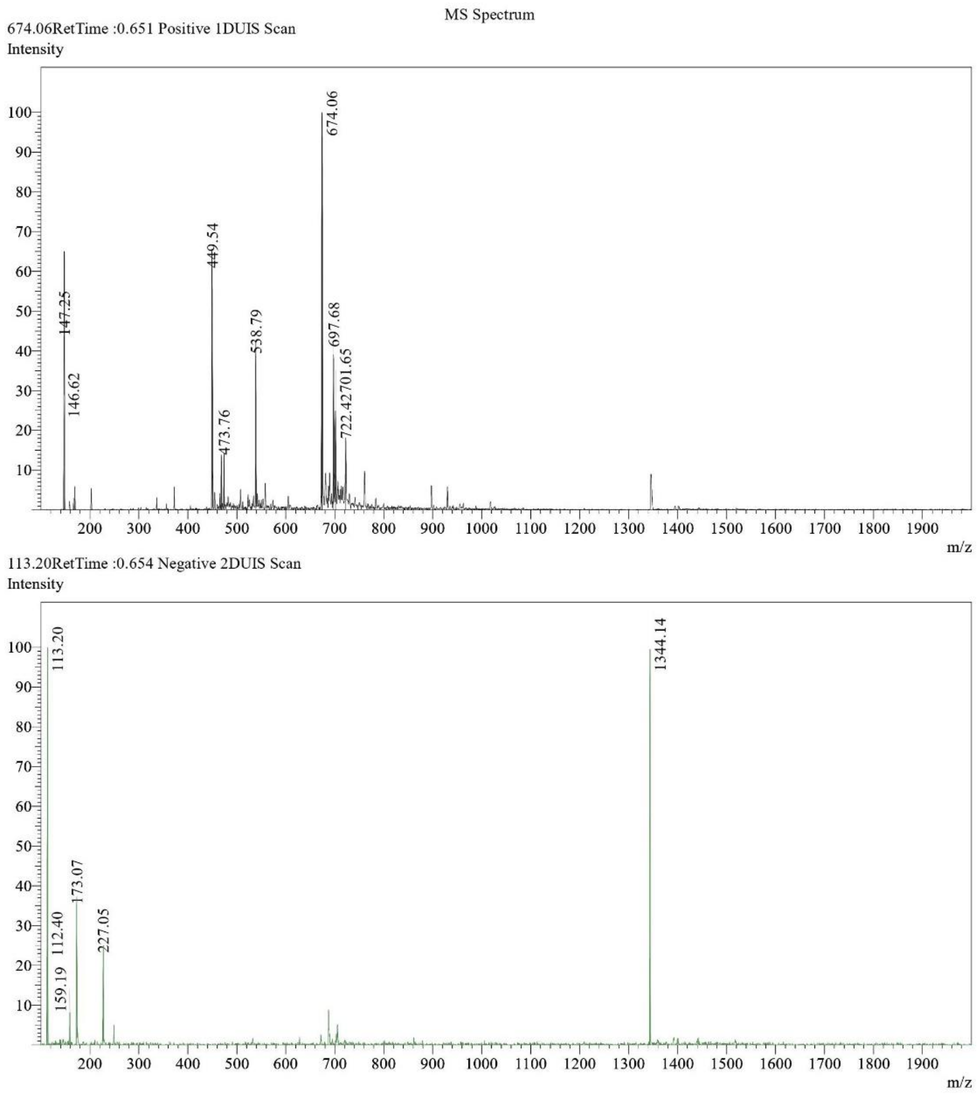

Figure 19. Mass Spectroscopy data of DEC-3 showing M-1 peak. (Molecular Wt: 1345.59gmol^{-1}.

Molecular Docking Study:

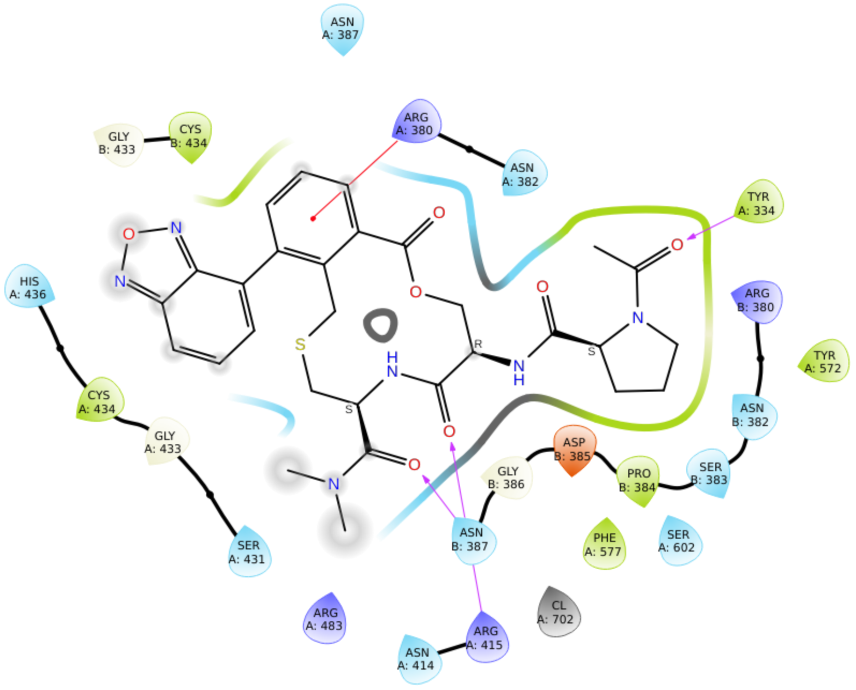

Figure 20. 2D interactions of reference molecule (91M) with Keap-1 (7Q6S)

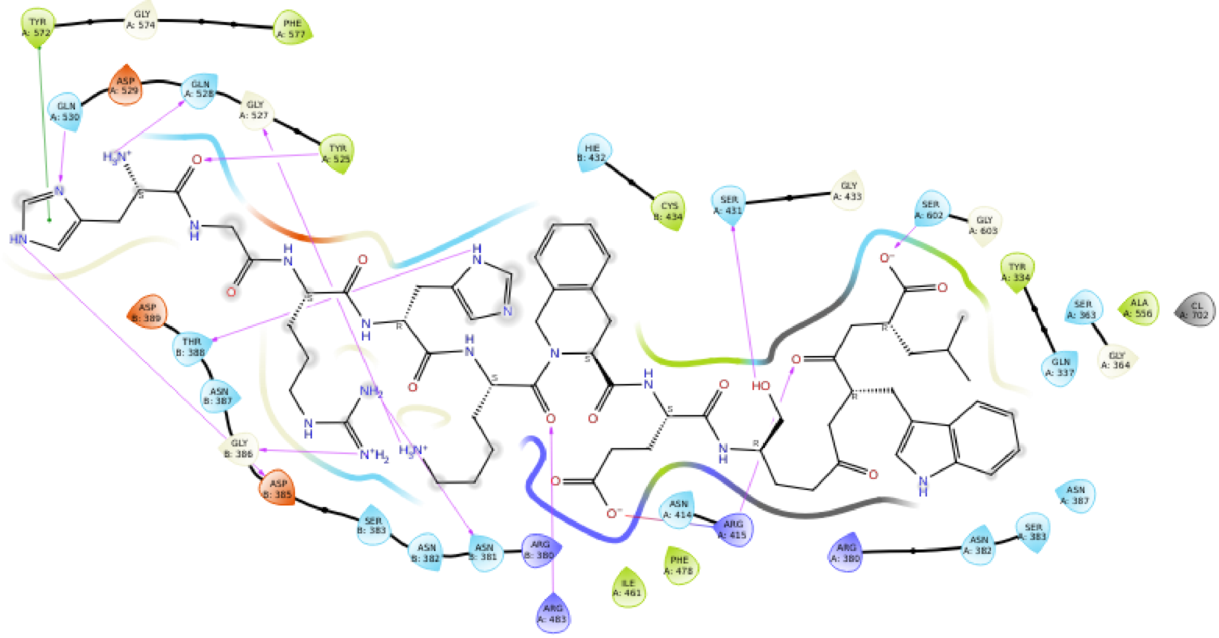

Figure 21. 2D interactions of DEC-1 with Keap-1 (7Q6S)

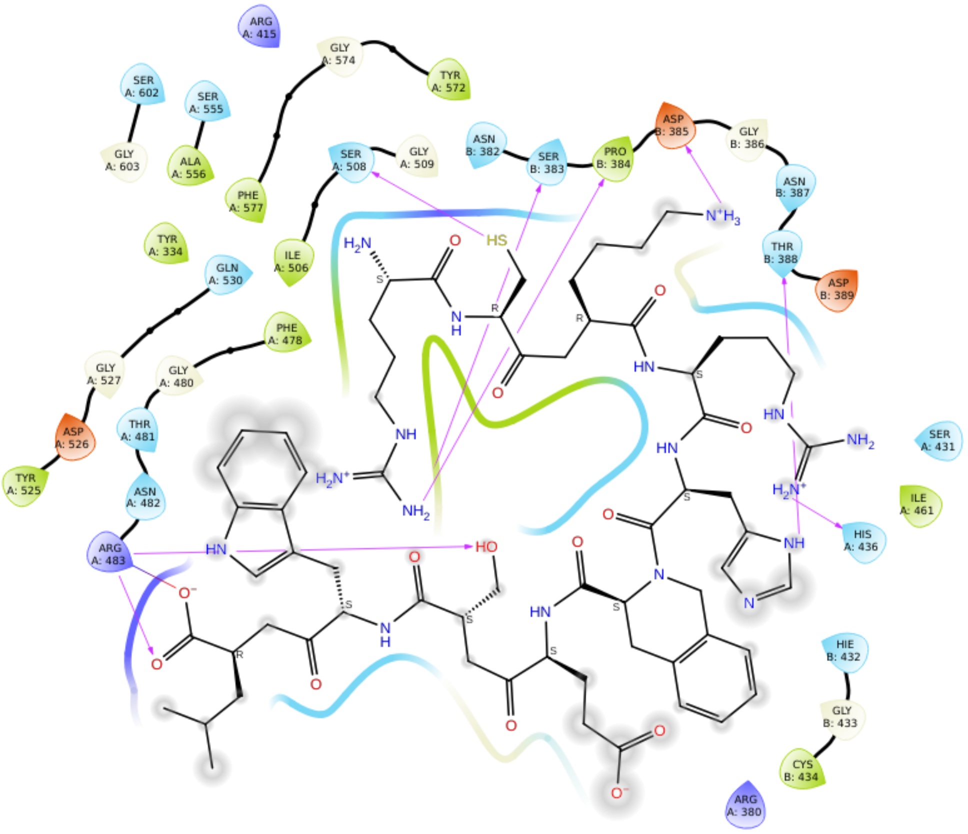

Figure 22. 2D interactions of DEC-2 with Keap-1 (7Q6S)

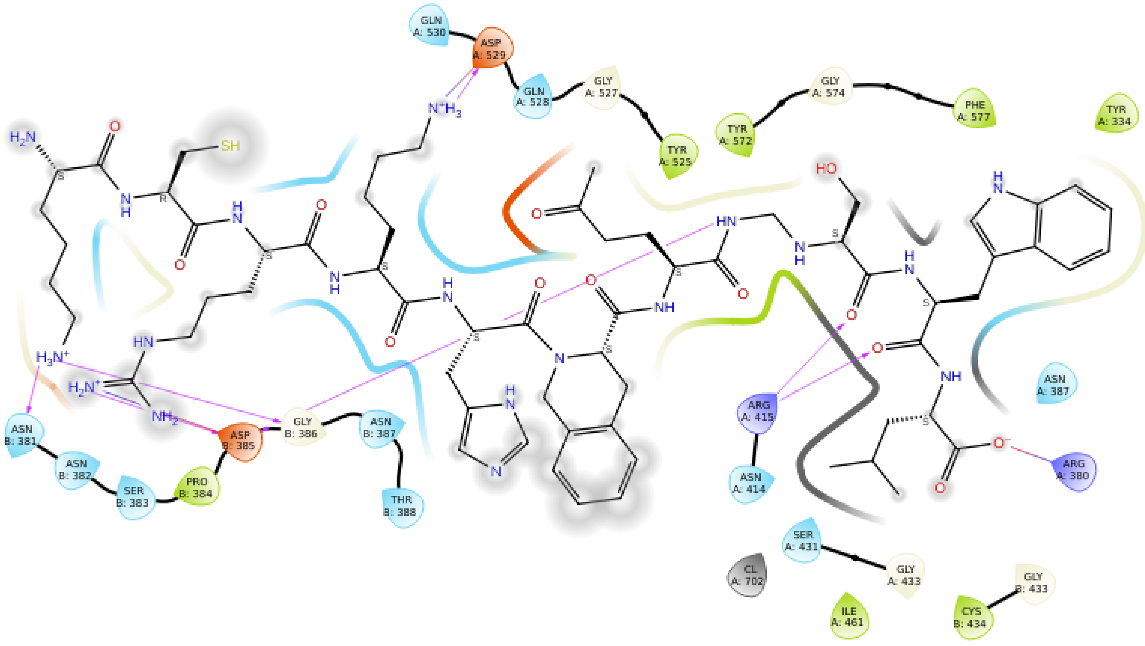

Figure 23. 2D interactions of DEC-3 with Keap-1 (7Q6S)

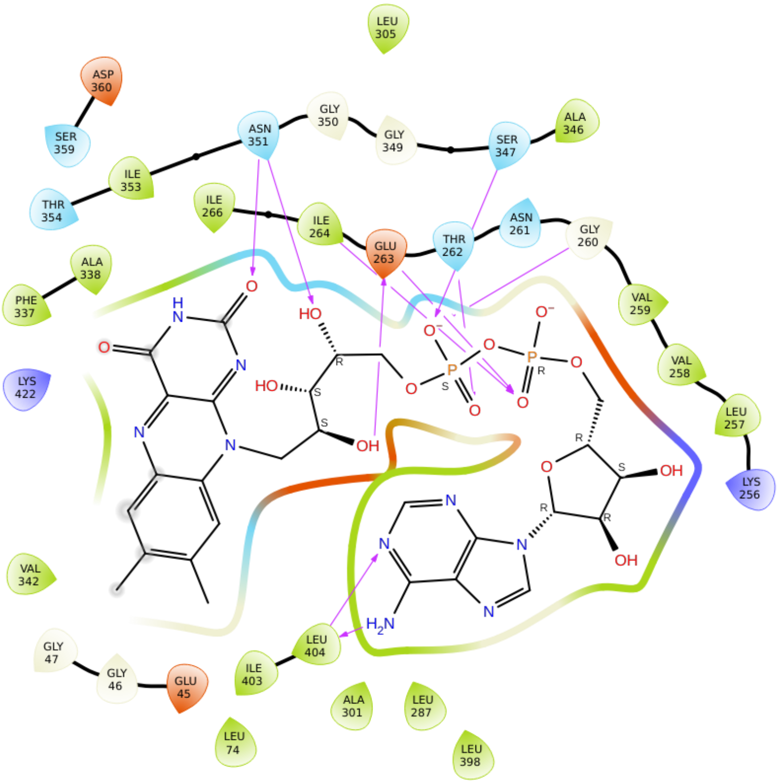

Figure 24. 2D interactions of reference molecule (FAD) with Xanthine Oxidase (2CKJ)

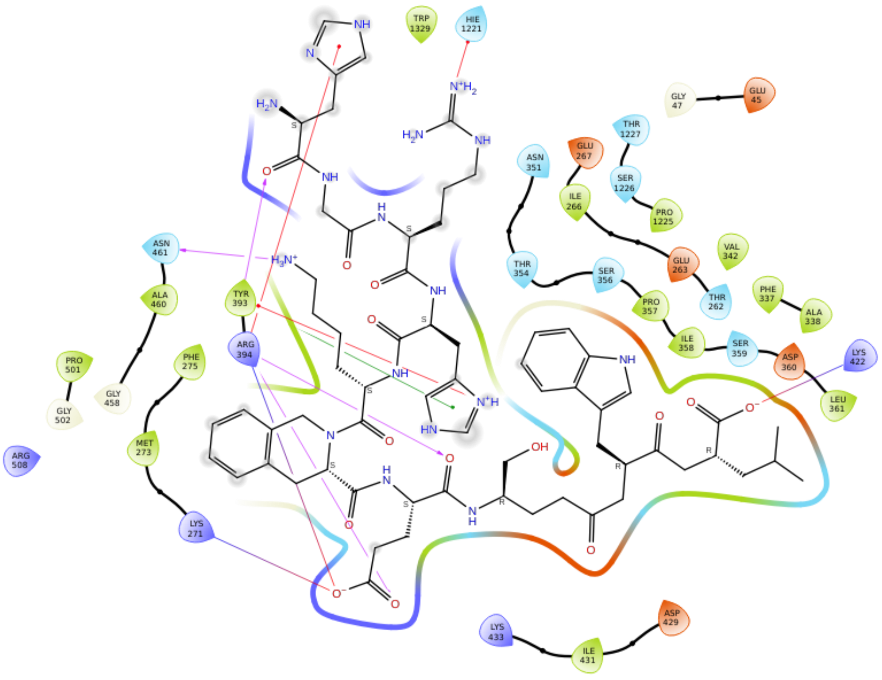

Figure 25. 2D interactions of DEC-1 with Xanthine Oxidase (2CKJ)

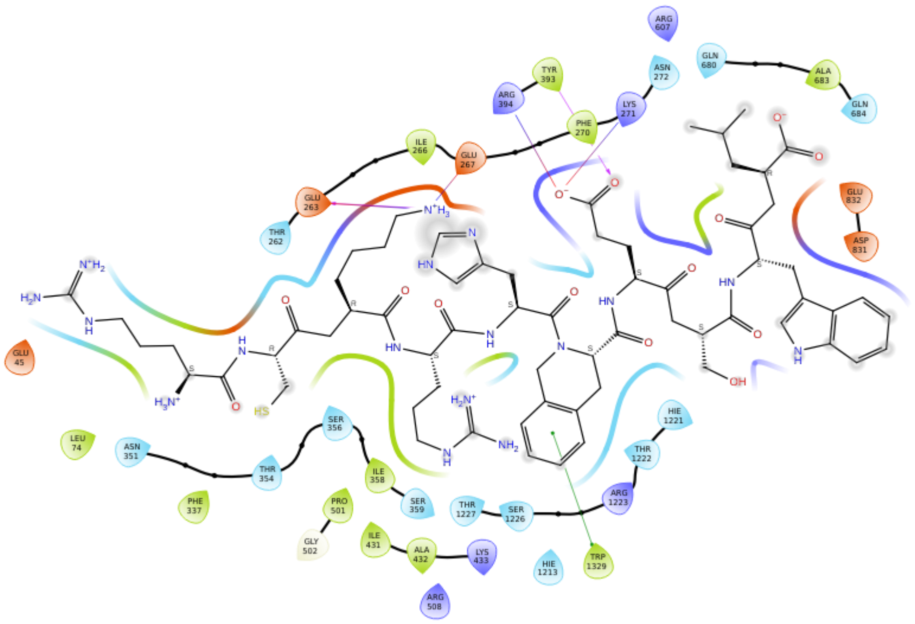

Figure 26. 2D interactions of DEC-2 with Xanthine Oxidase (2CKJ)

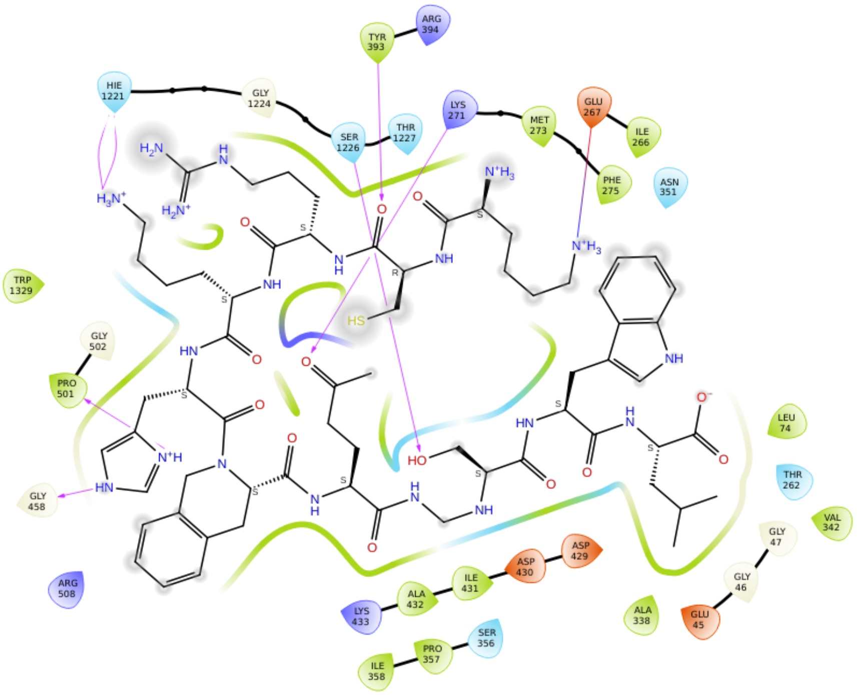

Figure 27. 2D interactions of DEC-3 with Xanthine Oxidase (2CKJ)
